# Inputs-Oriented VRS DEA in dairy farms

**DOI:** 10.12688/f1000research.132421.1

**Published:** 2023-07-28

**Authors:** C. A. Zuniga-Gonzalez, J. L. Jaramillo-Villanueva, N.E Blanco-Roa

**Affiliations:** 1 Agroecology, National Autonomous University of Nicaragua, Leon, Leon, Leon, 21000, Nicaragua; 2 Economy, Postgraduate College, Mexico, Puebla, Cholula, 72760, Mexico; 3 Animal Production, National Autonomous University of Nicaragua, Leon, Leon, Leon, 21000, Nicaragua

**Keywords:** Slack, Technical Efficiency, Scale Efficiency, Peers, Lambda

## Abstract

**Background:** This paper aims to examine the efficiency of Mexico’s dairy farms within its four regions of Tlaxcala Stated.

**Methods:** The Envelope Data Analysis (DEA) applied to the variable returns to a scale model (VRS) for the year 2020. Also, the results reveal that Tlaxcala’s dairy farm efficiency, on the other hand, was adversely influenced by three inputs (costs): cost of investment in livestock, the total annual cost for feeding, reproduction, diseases and treatments, preventive medicine, sanitation, milking, fuel, and total labor.

**Results:** The main findings are as follows, first, the mean efficiency for CRS, VRS, and efficiency scale was poor, below 50%. Second, 11 dairy farms were found that acted as relative pairs or reference points in the efficiency frontier. Third, excesses (slack) are estimated by identifying the farms that needed to reduce their costs to maintain the optimal level of milk production. Fourth, it observed that there were farms with very high slack; therefore, in the cost reduction projections they exceeded 50% of the original costs.

**Conclusions:** Finally, It concluded that based on the DEA estimates of the efficiencies indicators, discovered that the mean efficiencies of the constant and variable returns to scale, and efficiencies scale is relatively poor but significant in this production process. As part of the study, provided The Policy suggestions.

## Introduction

**Figure 1.**
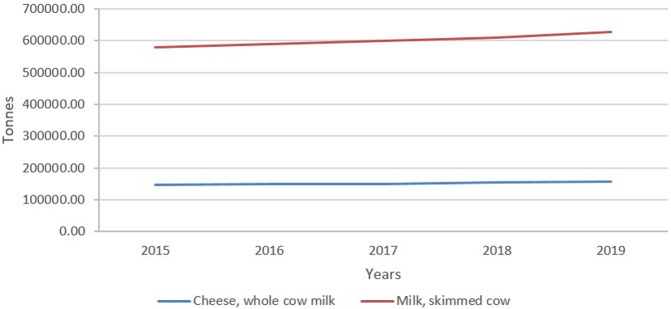
The Cheese, Whole cow milk and Milk, skimmed cow production, 2015–2019 (FAOSTAT).

Cattle farming is generally regarded as one of the most significant activities in Latin America due to its pronounced economic impact. ^1^^–^^3^ For this reason, the animal husbandry sector is the central axis of various performance evaluation studies and is where the analysis of technical efficiencies in this type of study becomes important. ^4^^,^^5^ The need for efficiency is not a new debate, but rather a concern that has its antecedents in Farrell. ^6^ The scientific community, producers, and policymakers are concerned about improving the efficiency and productivity of production, which is why they have decided to focus on rural development programs that seek to convert large-scale livestock production systems to intensive ones. Some plans to incorporate different strategies into their plans where efficiency and productivity variables are inherent. ^7^ Perez, ^1^ has measured that in America cattle practices positioned seventh in the world making and tenth in milk manufacture in 2001, which added about 7% to the world’s total meat production and 0.17% to milk. The problem is that it has not been possible to satisfy the requirements of demand, ^8^ meaning an imminent study of the efficiency of dual-purpose production systems in Latin America, where the tropics have great potential. Morrillo and Urdaneta ^9^ suggested that a farm that has cows will make 80% of their income from the milk and the last 20% from meat/grass/other products. ^10^ In any case, it’s influenced by the agroecological characteristics of the farm and the techniques used mainly depending on the grower’s goals, the stage of growth at which the males are sold, and the type of breed. ^11^ According to The Ministry of Agriculture and Rural Development of Mexico, in the State of Tlaxcala, the total economically active population employed 88.3% in agriculture and 11.7% in the livestock industry. Figure 1 depicts the increasing trend of Mexico’s Cheese, Whole cow milk, and Milk, skimmed cow production during 2015-2019. ^7^

According to the analysis of the 2013-2018 Sectoral Program for Agrarian, Fisheries and Nutritional Progress of Mexico, in 2050 the population will be 9.3 billion people and the Food and Agriculture Organization of the United Nations (FAO) estimates that the world demand for food will increase by 60%, this means that there will be more people to feed, provide housing, transportation, *etc.* For this reason, it is very important to assess whether productivity and efficiency grow at the rate that the population grows. ^12^ In Mexico between 1960 and 2021, the population has increased from 37.77 million to 126.71 million. ^1^ This represents an increase of 235.4% in 61 years. In relation to the problem of population growth and the capacity of governments to meet that demand, it is estimated that the population of the population of Mexico is predicted grow by three million during the year 2023, reaching 151 million. ^1^ The continued progress in some developing countries such as China, India and Brazil positions tasks and opportunities for the growth of the agri-food sector in order to meet the growing demand. Likewise, the International Monetary Fund projects that the world economy will grow at a compound annual rate of 3.8% over the next six years, with wide variations between groups of countries, of which 5.2% are in emerging markets and 2.2% in developed countries increasing global food consumption and trade. ^13^^,^^14^ This trend represents a huge opportunity for Mexico, which could play a leading role in gathering global food demand. However, the cultivable parcel is incomplete both in the world and in Mexico. We need to tackle climate change leading to extreme weather events affecting food production. In this context, increasing food making through increased efficiency is a major global challenge. In Mexico, climate change has emerged as an unexpected and unprecedented extreme. Regarding the rainfall regime, 2009 saw the greatest deficiency in 60 years and 2010 was the rainiest year on record. ^1^^,^^9^ In September 2013, heavy rains devastated agriculture and unfortunately claimed lives. In just a few days, several parts of the country received as much rain as in 2012. These regular sensations resulted in the loss of some production, the occurrence of disease, and the loss of earnings and prosperity of the people. The Mexican Climate Modeling Network has produced a series of projections that describe the country’s climate under different climate change scenarios. ^1^^,^^9^ There is consensus that overall temperature increases in Mexico over the next few decades will be 6% above the historical average and will exceed global temperature increases over the same period. ^1^^,^^9^

Therefore, an increased risk of climate events associated with rising temperatures or reduced agricultural production is expected in historically unrecorded locations. Most models do this for precipitation not taking into account the effects of tropical cyclones, northerly winds and hurricanes meaning the precipitation forecast is more uncertain. It is in this situation that learning about the effects of efficiency in livestock production systems is valuable as part of the livestock bioeconomy of the productive route of eco intensification. ^15^^–^^18^

Some works with a data envelope analysis (DEA) approach have been carried out in Latin America, such as the case of Arcos *et al.* ^19^ on milk production in the Ecuadorian mountain range, which represents 74% of the country. In the second phase of his research, he used the DEA model, which determined the efficiency of scale (EE) and the elasticities from 2014 to 2017 in each province.

In the same way, Sperat *et al.* ^20^ used DEA used on the grounds of data provided by the interviews carried out on each farm. Cluster analysis and discriminant analysis were completed in this study. The results show an efficiency level of 59.5% for the area and apparently, there are no indications to assume that certain production styles are a limiting factor for the productive potential of each farm.

The contribution of this article focused on the DEA study for efficiencies of Tlaxcala’s dairy farms, the mean efficiency for constant returns scale (CRS), variable returns scale (VRS), and the efficiency scale estimated. The DEA slack variable was directly related to problem-solving. The most productive and efficient dairy farms were identified, allowing the establishment of the efficiency frontier. This allowed estimating the slack of each dairy farm and then projecting the costs required to decrease in comparison with the benchmark or efficient pair. This was important for decision makers in the study region because it allowed identifying the causes of the low yields of efficiency and productivity in the area with the highest dairy production in Mexico.

This work is divided into a section related to a literature review of technical efficiency models, a third section devoted to the methodology (VRS) and scale efficiencies, a fourth sections to the empirical results, a fifth to a discussion (efficiencies measure VRS DEA model, slack measurements), and finally conclusions presented later.

## Literature review

The purpose of this section is to emphasize the importance of measuring efficiency and discuss how relative technological efficiency, often expressed as a frontier function, using several methods. The two methods that are the most commonly used: a) DEA, ^21^ which uses mathematical programming; and b) Stochastic frontier analysis (SFA), which uses econometric methods. ^7^ DEAP 2.1 software (RRID:SCR_023002). ^22^ For this study DEA methods were used.

Modern performance measurement Färe ^23^ started with Farrell ^6^ who improved on the work of Debreu ^24^ and Koopmans ^25^ to identify two components of efficiency in a decision-making unit (DMU). Technical efficiency, which represents a DMU’s ability to maximize its revenues through a range of inputs, and allocation efficiency, which is a DMU’s ability to optimize the ratio of inputs to changes in market prices. ^6^^,^^24^^,^^25^ Farrell’s initial idea was to identify the input space, so he derived an input reduction approach and an input-oriented measure.

### Slack

The DEA slack variable (λ) directly was related to problem-solving (see equation 3). For example, if the model finds that the optimal solution to the problem given by the model has an efficiency of one (perfect efficiency or Frontier), this means that the DMU being evaluated is efficient compared to other DMUs. Otherwise, the closer it gets to zero, the more inefficient it is. So, it can be said that efficiency is measured from 0 to 1. Summarizing, when closer to one (Efficiency Frontier [ϕ]) the DMU will be efficient and when closer to zero the DMU will be inefficient.

On the other hand, to measure slack, or the value necessary to reach the frontier, the relative efficiency value of the DMU is subtracted from 1 (value between 0-1), indicating the distance or level of costs that the DMU must reach to have optimum efficiency (Frontier). ^26^ Therefore, each DMU with an efficiency of one has a slip value equal to zero and a DMU with a Slack score means massive inefficiencies. Therefore, the higher the Slack score, the less efficient the evaluated DMU will be. ^27^

Researchers have experienced rapid growth in the use and theoretical scope of DEA ^28^^,^^29^ since its appearance in 1978 through the work of Farrell, ^6^ and Charnes. ^30^ The objective of the present study was to provide the measurement of the costs of inputs used in the production process (input) and the income (output) of the different DMUs. Each relative efficiency is assigned a quantification or measurement value. The output in terms of highest income achieves the lowest possible input in terms of costs and in this sense the efficiency frontier is determined.

Two strategies were used to estimate the above efficiencies, depending on whether they were input or output oriented. ^31^ The first CRS/VRS model, ^31^^,^^32^ input-oriented, is concerned with achieving the maximum proportional reduction of the input vector given the level of the output while keeping the limit or level of the output constant. Output-oriented models, on the other hand, consider a given input and aim for the maximum proportional increase in output while staying within possible limits.

## Methods

### The variable returns to scale model (VRS) and scale efficiencies

This study used an input-oriented multilevel DEA VRS model to process input slack variables and run a series of radial linear programming (LPs) to identify predicted efficiency points. ^32^ The inputs-oriented technical efficiency measure addresses the question: “By how much can input values be proportionally reduced without changing the output values produced”. In the study this means measuring and identifying the farms that manage to reduce their costs while maintaining the same level of milk production.

The sample consisted of 102 observations for one output (y) and three inputs (x _1,_ x _2_, x _3_). They were taken from six region of the Tlaxcala stated. The regions were selected by statistical criteria of conglomerates in such a way that the sample was homogeneous and statistically significant. For the data, a questionnaire with 42 variables was elaborated with the purpose of carrying out a socio-economic diagnosis and measuring the efficiency and productivity of the production units. For the measure on the efficiency and productivity, only three were selected (input-output), which are required for the purpose of the investigation. ^33^

DEA is a nonparametric mathematical programming estimation focused on the computation of limits y. A research unit is a decision unit of the DMU. ^2^^,^^22^^,^^34^^–^^37^ Since DEA is best presented by percentage or ratio, it need to get the percentage of all outputs to all inputs so that u’ y _i_ /v’ x _i_ can be plotted, where u is an M-by-1 vector of output weights, v is a K-by-1 vector of input weights or proportions. ^7^ Then the u’ y _i_/v’ x _i_ represents the efficiency (ϕ) measured as a percentage. The following Banker Charnes Cooper (BCC) mathematical programming model ^32^ is specified to select the optimal weights or proportions ( Equation 1):

$$\begin{array}{c} \max_{u,v}\left(u^{\prime }y_{i}/v^{\prime }x_{i}\right),\\ s.t\;\frac{u^{\prime }y_{i}}{v^{\prime }x_{i}}\leq 1,J=1,2,\ldots \ldots \ldots \ldots .,N\\ u,v\geq 0 \end{array}$$ (1)

The resulting values of u and v represent the efficiency measure for each maximized DMU, subject to the constraint that all measures must be greater than or equal to one problem with this estimate is that there are infinitely many solutions. To avoid this, a restriction is proposed considering v’ x _i_=1, J represents the number of each farm dairy selected ( Equation 2): ^32^

$$\begin{array}{c} \max_{u,v}\left(\frac{u^{\prime }y_{i}}{v^{\prime }x_{i}}\right),\\ s.t\;\frac{u^{\prime }y_{i}}{v^{\prime }x_{i}}=1,\\ \begin{array}{c} u^{\prime }y_{i}-v^{\prime }x_{i}\leq 0,J=1,2,\ldots \ldots \ldots \ldots .,N\\ u,v\geq 0 \end{array} \end{array}$$ (2)

Note that the expressions for u and v have changed. Were expanded to the shape is unknown in the way the multipliers in the linear programming problem posed. Therefore, using duality in linear programming, we can derive the equivalent form ( Equation 3). This is the case when the CRS linear programming problem can be easily modified to account for VRS by adding the convexity constraint ^32^: $N1^{\prime }\lambda =1$ . Where θ is the Efficiency coefficients. $y_{i}$ represents the output and $x_{i}$ the inputs. Finally, $\left(\lambda \right)$ represents the Slack in percentage, so it is the necessary value for reach the frontier.

$$\begin{array}{c} \min_{,\theta ,\lambda }\left(\theta \right),\\ s.t-y_{i}+\mathrm{Y\lambda}\geq 0,\\ \begin{array}{c} \theta x_{i}-\mathrm{X\lambda}\geq 0,\\ N1^{\prime }\lambda =1\\ \lambda \geq 0. \end{array} \end{array}$$ (3)

Equation 3 denotes the case of N1 that would be a vector N x1. It represents an enclosing or expansion form that minimizes the restrictions imposed by the multiplier form (KM < N 1) and is the preferred way of finding the solution according to Farrell. ^6^ This equation allows us to convert from CRS to VRS. This is because cross-efficiency evaluation in DEA has been developed under the assumption of CRS, and no valid attempts have been made to apply the cross-efficiency concept to the VRS condition. This is due to the fact that negative VRS cross-efficiency arises for some DMUs. Since there exist many instances that require the use of the VRS DEA model, it is imperative to develop cross-efficiency measures under VRS. The value of θ represents an estimate of the efficiency measure for each DMU. This is true for θ ≤ 1 according to Farrel, ^6^ Lanteri ^35^ and Shephard. ^38^ Here, a value of one indicates a cut-off point and therefore an efficiency measure for each DMU. This way, the efficiency (ϕ) and slack (λ) were estimated for each dairy farm. To run DEAP 2.1 software it is necessary to use three files. The first is the data file where the data of the variables built is located in the order Output, input 1, input 2 and input 3. The second file is the instructions file where it is specified that there are 102 observations (n), one output, and three inputs, the DEA orientation and the assumed scale that in the study is VRS.

### Data source and location

The study was conducted in the state of Tlaxcala, located in the highlands of Mexico. The geographic coordinates are 98 degrees 3 inches west longitude, 19 degrees north latitude, 97 degrees 38 minutes north latitude, 19 degrees north latitude, and 06 degrees latitude. The state’s general climate is mild with some rain in the summer. The typical elevation of the revision area is 2,200 meters above sea level. The cluster technique was applied, carrying out the following steps to carry out a cluster sampling:

[a] Dairy farms were defined as the target population.
[b] The desired sample size to carry out the statistical study is determined
[c] The clusters were defined for this purpose, four types of farms were identified. The four type are defined by the size of each farm. Cesin-Vargas ^39^ and Cuevas Reyes ^40^ mention that in the study area there are four types of dairy farms, added through the use of principal components, cluster analysis and analysis of variance, four types of livestock farms were identified and characterized; small cattle farms (67%), medium cattle farms (24%), large cattle farms (7%), and large cattle farms with business potential (2%). For the purposes of this study, we worked with the typology of small livestock farms.
[d] The clusters that formed the sample of the statistical study were randomly selected.

The data collection procedure was as follows:

[a] The questionnaire was designed keeping in mind that it would be used for various purposes, such as socioeconomic diagnosis, efficiency and productivity analysis with the DEA approach, and efficiency analysis with the SFA approach. Consequently, of the 40 variables collected, only one output and three inputs were used. And of the 118 randomly visited dairy farms, only 102 met the statistical selection criteria.
[b] The collected data were entered into a database built with the IBM SPSS Statistics program (RRID: SCR_016479) v.22.
[c] The variables to be used in this study were selected. For this, the output variable was built by adding Total annual sale (USD) and Total annual sale of products obtained on the farm (USD).
[d] Input 1 was built from the variable Cost of investment in livestock (USD), input 2 was built by adding the variables Annual cost of fuel (USD), plus Annual cost of food (USD, plus Annual cost of concept of reproduction (USD, plus annual cost for animal health (USD). And input 3 was built by the variables Total annual cost of labor (USD), including hired labor and family labor.
[e] With the variables built (Output, and its three inputs) it was transferred to the database required by the DEAP 2.1 software (RRID:SCR_023002) transferring to the file data file format included in the software.

The processing of the data was similar in other studies, however the ordering and processing of the information is different. The software DEAP 2.1 consider tree file: datafile, instruction file and output or results. In this study, the data was organized using the DEA approach. ^33^^,^^41^

### Sample size and variables

In 2020, the study was performed. The sample number (n) was 102 dairy farms in six communities or regions across the Tlaxcala stated. The sample was estimated according to equation 4 where the population was 71,000 farms according to the Secretary of Agricultural and Livestock Information (SIAP), ^10^ the Z is a parameter estimated of 1.93 (see Table 1) that was estimated with a probability (p) of 50% as well as (q) 50% and the margin of error of 9%. Of the 118 that were estimated according to the formula of equation 4, only 102 were worked on since the rest were not statistically significant for the objective of this investigation. The production units were selected randomly and distributed among the six regions of the stated of Tlaxcala that are important for milk production. The criteria for making the selection were: first, randomly selected, that is, that all the subjects of the population of dairy farms had the same possibility of being selected in this sample and therefore being included in the study; and secondly, that the number of selected dairy farms numerically represent the population that gave rise to it with respect to the distribution of the variable under study in the population, that is, the estimation or calculation of the sample size. For this, the following formula ^42^ was used:

$$n=\frac{N*z_{\alpha }^{2}*p*q}{e^{2}*\left(N-1\right)+z_{\alpha }^{2}*p*q}$$ (4)

Where,

*n* Sample size

*N* Population size

*Z* Statistical parameter on which *N* depends (95% = 1.96)

*p* Probability of the event occurring (50%)

*q* Represents (1- *p*) probability that the event will not occur (50%)

*e* Maximum accepted estimation error (9%)

**Table 1.** Z score for the *p-*value and confidence level. ^44^

Z-score (Standard deviation)	p-value (Probability)	Confidence level
<-1.65 or > +1.65	<0.10	90%
< -1.96 or > +1.96	<0.05	95%
< -2.58 or > +2.58	<0.01	99%

### Variables

This study used the DEAP 2.1 software (RRID:SCR_023002) ^22^ on a computer ^32^^,^^41^^,^^43^ to get standard CRS and VRS DEA model that involve the calculation of technical and scale efficiencies ^31^^,^^32^ of the data sampled during the study period 2020. ^23^ This program involves a simple batch file system where the user creates a data file and small file containing instructions. The files are available in Zuniga and Jaramillo. ^33^ The text to file data refer to S3, ^33^ contains 102 observation on one output and tree inputs. The output “Total income (USD)” is listed in the first column and the inputs “Cost of investment in livestock (USD)”,“Total annual cost for feeding”, “reproduction”, “diseases and treatments”, “preventive medicine”, “sanitation”, “milking”, “fuel (USD)” and “Total labour (USD)”.

Output (TVA _i_) signifies the total annual sale of products obtained on the farm, such as the amount of milk produced per cow per year and by secondary products. The unit of measure is in USD USA. ^7^

Input 1 (CIG _ij_) signifies the annual value of the cattle investment quantified in USD USA.

Input 2 (CT _ij_) signifies the total annual cost for fuel, feeding, reproduction, illness and treatment, milking, mortality, and preventive medicine, measured in annual USD. ^7^

Input 3 (MO _ij_) Signifies the annual cost of family and hired labor, measured in USD.

Table 2 displays the descriptive statistics for the model’s variables. Revenue from sales of milk and by-products (TVA) during the study period on average was 3.8 million USD, with a standard deviation of 1.8 million USD. The costs for investment in the cattle herd inventory on average was 1.0 million USD, with a standard deviation of 440.1 thousand USD. In the case of the costs of fuel, food, veterinary treatment and other inputs, the average cost was 1.0 million USD with a standard deviation of 494 thousand USD per year, and finally the average cost of labor was 235 thousand USD per year with a standard deviation of 37 thousand USD. All statistical analysis was completed using the IBM SPSS Statistics (RRID: SCR_016479) v.22. The full protocol can be found on protocols.io. ^45^

**Table 2.** Descriptive statistics of the variables.

Variables	(TVA _i_)	(CIG _ij_)	(CT _i_j)	(MO) _ij_
Statistics				
N	102	102	102	102
Minimum	22300	16000	7300	43800
Maximum	156103200	38826000	44020690	2701000
Mean	3852213.65	1028312.75	1029632.73	235168.33
Standard Deviation	18148129.586	4401851.614	4942898.582	377025.731

## Results and discussion

The results were obtained following the DEA BCC model ( equation 1-3), identifying the most efficient (ϕ) dairy farms and in this way it is quantified how many times they are a reference for dairy farms that did not reach the efficiency frontier (ϕ), adding the percentages of their costs to reduce to reach the optimal level, this is the slack (λ).

### Efficiencies measure VRS DEA model

Table 3 displays the findings for Mexico and its 102 dairy farms based on the VRS and scale efficiencies estimations that involve the calculation of technical and scale efficiencies for the estimation of the efficiency (ϕ) and the slack (λ). ^46^ The results constructed on the methodology of Färe *et al.*, ^23^ and Banker, Charnes, and Cooper ^32^ to account for VRS. ^27^^,^^28^^,^^46^ The use of the VRS specification permits the calculation of technical efficiency from CRS DEA, technical efficiency from VRS DEA, and scale efficiency equal crste/vrste (constant return scale technical efficiency between variable return scale technical efficiency). Farm number 1, 56, and 75 are efficient under both CRS and VRS technologies. The VRS technical efficiency (TE) is equal to 1 on farms number 6, 8, 36, 41, 53, 86, 90, 91, 92 and 93 also showing the increasing returns to scale (IRS) portion of the VRS frontier. In general, the mean efficiencies for CRS were 25%, VRS 53%, and scale efficiency 44%.

**Table 3.** Efficiency summary.

farm	crste	vrste	scale		farm	crste	vrste	scale	
1	1	1	1	-	52	0.686	0.794	0.864	irs
2	0.04	0.224	0.179	irs	53	0.572	1	0.572	drs
3	0.588	0.714	0.823	irs	54	0.423	0.762	0.556	drs
4	0.291	0.403	0.722	irs	55	0.724	0.792	0.915	irs
5	0.375	0.736	0.509	irs	56	1	1	1	-
6	0.683	1	0.683	irs	57	0.434	0.836	0.519	drs
7	0.384	0.51	0.753	irs	58	0.512	0.594	0.861	irs
8	0.411	1	0.411	irs	59	0.108	0.593	0.182	irs
9	0.324	0.396	0.817	irs	60	0.117	0.208	0.562	irs
10	0.316	0.862	0.367	irs	61	0.174	0.38	0.456	irs
11	0.349	0.879	0.397	irs	62	0.04	0.233	0.171	irs
12	0.477	0.669	0.713	irs	63	0.425	0.8	0.532	drs
13	0.34	0.447	0.761	irs	64	0.064	0.229	0.277	irs
14	0.41	0.533	0.769	irs	65	0.161	0.269	0.598	irs
15	0.278	0.346	0.803	irs	66	0.06	0.207	0.29	irs
16	0.073	0.526	0.138	irs	67	0.036	0.392	0.091	irs
17	0.36	0.998	0.361	irs	68	0.023	0.14	0.165	irs
18	0.049	0.232	0.212	irs	69	0.089	0.278	0.319	irs
19	0.115	0.21	0.55	irs	70	0.093	0.179	0.522	irs
20	0.038	0.125	0.304	irs	71	0.417	0.612	0.681	irs
21	0.061	0.129	0.475	irs	72	0.075	0.214	0.35	irs
22	0.431	0.566	0.762	irs	73	0.456	0.622	0.733	irs
23	0.614	0.963	0.638	irs	74	0.09	0.239	0.377	irs
24	0.028	0.205	0.137	irs	75	1	1	1	-
25	0.056	0.212	0.262	irs	76	0.565	0.901	0.627	irs
26	0.04	0.136	0.293	irs	77	0.484	0.691	0.7	irs
27	0.088	0.284	0.31	irs	78	0.069	0.222	0.311	irs
28	0.105	0.557	0.188	irs	79	0.064	0.281	0.229	irs
29	0.104	0.321	0.325	irs	80	0.13	0.138	0.947	drs
30	0.048	0.363	0.134	irs	81	0.066	0.153	0.431	irs
31	0.141	0.146	0.96	irs	82	0.087	0.156	0.555	irs
32	0.072	0.635	0.113	irs	83	0.089	0.183	0.487	irs
33	0.098	0.275	0.356	irs	84	0.067	0.806	0.083	irs
34	0.116	0.683	0.169	irs	85	0.074	0.142	0.521	irs
35	0.071	0.368	0.192	irs	86	0.316	1	0.316	irs
36	0.281	1	0.281	irs	87	0.054	0.76	0.071	irs
37	0.062	0.32	0.194	irs	88	0.491	0.816	0.601	irs
38	0.077	0.236	0.327	irs	89	0.485	0.604	0.803	irs
39	0.259	0.345	0.751	irs	90	0.04	1	0.04	irs
40	0.064	0.744	0.086	irs	91	0.674	1	0.674	irs
41	0.157	1	0.157	irs	92	0.211	1	0.211	irs
42	0.097	0.422	0.23	irs	93	0.05	1	0.05	irs
43	0.098	0.575	0.17	irs	94	0.054	0.591	0.091	irs
44	0.068	0.327	0.209	irs	95	0.34	0.489	0.696	irs
45	0.083	0.439	0.189	irs	96	0.067	0.727	0.092	irs
46	0.317	0.433	0.731	irs	97	0.054	0.826	0.065	irs
47	0.066	0.199	0.333	irs	98	0.03	0.381	0.08	irs
48	0.083	0.357	0.234	irs	99	0.025	0.215	0.117	irs
49	0.287	0.595	0.482	irs	100	0.318	0.838	0.379	irs
50	0.067	0.266	0.252	irs	101	0.472	0.875	0.539	irs
51	0.563	0.583	0.965	irs	102	0.244	0.514	0.473	irs
					mean	0.245	0.531	0.441	

Source: Author's calculation.

### Slack measure (λ)

Koopmans’s ^25^ definition of technical efficiency was stricter than Farrell’s ^6^ definition. Thus, Koopmans argued that both Farrell’s measure of technical efficiency and any non-zero input slack reported providing an accurate indication of the technical efficiency of a farm dairy in DEA analysis. Input slack is also known as input overload in some literature. ^47^ Table 4 presents the % weight peers and summary lambda (λ). The peer represents the dairy farm that reached the efficiency frontier (ϕ) in terms of costs and income, and the number of times that they serve as a peer to others. Slack’s estimations (λ) represent the cost that each dairy farm must be reduced to reach an efficient point. On the other hand, Slack’s estimations (λ) based on Ali and Seiford ^48^ using second-stage linear programming to consider the cost that must be reduced to reach the level of the efficiency frontier. ^49^ The values inside the parentheses are given in percentages and represent the slack or excess of the input that should be multiplied by values shown in Tables 6, 7 and 8 the values outside the parentheses are peers for the evaluated farm. Table 5 shows the number times each farm is a peer to another. It can be noted that farms Numbers 6, and 75 (peer count 62) are the ones that are most often peers, that is, their costs mark the efficiency frontier to be followed by the other farms that are outside.

**Table 4.** Summary Peers and lambda weigh %.

farm	Peer	(λ)			farm	Peers	(λ)		
1	1 (1)				52	56 (0.009)	6 (0.088)	91 (0.903)	
2	75 (0.011)	90 (0.707)	6 (0.282)		53	53 (1)			
3	56 (0.001)	1 (0.282)	6 (0.717)	75 (0)	54	75 (0.202)	56 (0.798)		
4	1 (0.263)	86 (0.026)	6 (0.711)		55	56 (0.74)	36 (0.26)		
5	91 (0.275)	6 (0.49)	92 (0.235)		56	56 (1)			
6	6 (1)				57	75 (0.049)	56 (0.951)		
7	56 (0.001)	1 (0.049)	91 (0.95)		58	56 (0.003)	1 (0.043)	6 (0.954)	75 (0)
8	8 (1)				59	75 (0.015)	6 (0.446)	90 (0.363)	93 (0.176)
9	56 (0.001)	1 (0.308)	91 (0.072	6 (0.62)	60	75 (0.086)	86 (0.363)	6 (0.306)	93 (0.246)
10	92 (0.609)	91 (0.352)	8 (0.038)		61	75 (0.053)	41 (0.237)	93 (0.71)	
11	6 (0.218)	1 (0.047)	86 (0.735)	75 (0)	62	6 (0.528)	75 (0.01)	90 (0.462)	
12	56 (0)	1 (0.096)	91 (0.904)		63	75 (0.938)	56 (0.062)		
13	56 (0.003)	91 (0.877)	6 (0.121)		64	75 (0.04)	86 (0.028)	6 (0.47)	93 (0.462)
14	56 (0.002)	91 (0.537)	6 (0.46)		65	6 (0.549)	75 (0.108)	90 (0.343)	
15	56 (0.001)	1 (0.202)	6 (0.797)	75 (0)	66	6 (0.676)	75 (0.027)	90 (0.298)	
16	75 (0.01)	41 (0.276)	90 (0.714)		67	75 (0.004)	90 (0.932)	6 (0.065)	
17	91 (0.995)	36 (0.005)			68	75 (0.009)	6 (0.678)	90 (0.095)	93 (0.218)
18	6 (0.332)	75 (0.015)	90 (0.652)		69	75 (0.046)	93 (0.569)	86 (0.321)	6 (0.064)
19	75 (0.036)	90 (0.108)	6 (0.856)		70	75 (0.064)	6 (0.717)	90 (0.105)	93 (0.115)
20	75 (0.014)	86 (0.016)	6 (0.785)	93 (0.186)	71	91 (0.875)	1 (0.044)	6 (0.081)	
21	6 (0.768)	75 (0.047)	90 (0.185)		72	75 (0.041)	93 (0.13)	6 (0.3)	90 (0.529)
22	56 (0.002)	1 (0.011)	91 (0.959)	6 (0.029)	73	75 (0.158)	90 (0.842)		
23	56 (0.002)	36 (0.079)	91 (0.92)		74	75 (0.055)	6 (0.373)	90 (0.284)	93 (0.287)
24	6 (0.816)	90 (0.184)			75	75 (1)			
25	90 (0.979)	75 (0.021)			76	91 (0.746)	92 (0.073)	6 (0.181)	
26	6 (0.407)	75 (0.027)	90 (0.566)		77	56 (0)	1 (0.051)	91 (0.949)	
27	6 (0.6)	75 (0.03)	90 (0.37)		78	75 (0.026)	93 (0.04)	6 (0.728)	90 (0.205)
28	75 (0.012)	90 (0.633)	6 (0.354)		79	75 (0.02)	93 (0.108)	6 (0.508)	90 (0.363)
29	6 (0.834)	75 (0.015)	90 (0.151)		80	1 (0.876)	75 (0.119)	56 (0.005)	
30	75 (0.005)	6 (0.683)	90 (0.312)		81	75 (0.068)	93 (0.266)	6 (0.336)	90 (0.329)
31	56 (0.001)	75 (0.098)	6 (0.901)		82	75 (0.09)	90 (0.373)	6 (0.537)	
32	75 (0.008)	93 (0.688)	86 (0.062)	6 (0.242)	83	75 (0.068)	90 (0.453)	6 (0.48)	
33	6 (0.575)	75 (0.038)	90 (0.387)		84	41 (0.482)	75 (0.001)	90 (0.517)	
34	75 (0.015)	41 (0.253)	90 (0.732)		85	75 (0.06)	6 (0.723)	90 (0.062)	93 (0.155)
35	75 (0.018)	93 (0.761)	90 (0.004)	6 (0.217)	86	86 (1)			
36	36 (1)				87	41 (1)			
37	75 (0.021)	86 (0.575)	93 (0.404)		88	91 (0.829)	8 (0.02)	6 (0.151)	
38	75 (0.049)	93 (0.414)	6 (0.415)	90 (0.122)	89	56 (0.003)	1 (0.003)	91 (0.973)	6 (0.021)
39	56 (0.001)	1 (0.002)	91 (0.997)		90	90 (1)			
40	6 (0.114)	90 (0.133)	75 (0.004)	93 (0.749)	91	91 (1)			
41	41 (1)				92	92 (1)			
42	90 (0.38)	6 (0.603)	75 (0.017)		93	93 (1)			
43	75 (0.012)	6 (0.37)	90 (0.521)	93 (0.097)	94	6 (0.457)	75 (0.004)	90 (0.148)	93 (0.39)
44	6 (0.487)	75 (0.015)	90 (0.499)		95	1 (0.384)	6 (0.275)	75 (0)	86 (0.341)
45	75 (0.015)	86 (0.05)	93 (0.934)		96	75 (0.001)	93 (0.329)	90 (0.214)	41 (0.455)
46	56 (0.001)	1 (0.035)	91 (0.964)		97	41 (0.461)	90 (0.539)		
47	90 (0.301)	6 (0.665)	75 (0.034)		98	90 (0.182)	6 (0.294)	93 (0.521)	75 (0.004)
48	90 (0.377)	6 (0.605)	75 (0.018)		99	75 (0.008)	93 (0.369)	6 (0.425)	90 (0.198)
49	6 (0.158)	75 (0.061)	90 (0.781)		100	8 (0.244)	92 (0.363)	91 (0.393)	
50	6 (0.785)	75 (0.013)	90 (0.202)		101	56 (0.002)	36 (0.255)	91 (0.743)	
51	56 (0.032)	1 (0.02)	91 (0.948)		102	86 (0.272)	6 (0.728)	93 (0)	

Source: Author's calculation.

**Table 5.** Peer count summary.

farm	peer count *
1	17
6	62
8	3
41	7
56	23
75	62
86	11
90	46
91	21
92	4
93	26

* Number of times each farm is a peer for another.

**Table 6.** Projection summary.

Farm	Input	Original movement	Radial movement	Slack Value	Projected	Farm	Input	Original movement	Radial movement	Slack value	Projected
1	1	75600	0	0	75600	22	1	116000	-50380.965	0	65619.035
	2	29542	0	0	29542		2	84207	-36572.672	0	47634.328
	3	44	0	0	44		3	5	-2.172	0	2.828
2	1	92000	-71395.164	0	20604.836	23	1	159200	-5888.03	-78671.632	74640.338
	2	126380	-98075.227	-11154.425	17150.348		2	62139	-2298.218	0	59840.782
	3	240900	-186946.69	0	53953.314		3	2	-0.074	0	1.926
3	1	66000	-18850.643	0	47149.357	24	1	109800	-87276.256	0.591	22524.335
	2	43509	-12426.858	0	31082.142		2	1026620	-816025.05	-190064.98	20529.971
	3	96	-27.419	0	68.581		3	65700	-52222.678	0.354	13477.676
4	1	109200	-65164.668	-5424.745	38610.587	25	1	96200	-75823.259	0	20376.741
	2	59565	-35545.178	0	24019.822		2	117785	-92836.201	-9173.578	15775.221
	3	58	-34.611	0	23.389		3	591300	-466052.94	-49436.191	75810.87
5	1	62000	-16341.696	-9426.539	36231.765	26	1	181200	-156518.58	0	24681.421
	2	35859	-9451.562	0	26407.438		2	176120	-152130.53	-4160.806	19828.664
	3	12	-3.163	0	8.837		3	343440	-296659.72	0	46780.283
6	1	24000	0	0	24000	27	1	95000	-68046.636	0	26953.364
	2	22120	0	0	22120		2	202086	-144750.24	-35463.697	21872.067
	3	15	0	0	15		3	116800	-83661.548	0	33138.452
7	1	132200	-64831.885	-5054.744	62313.37	28	1	38400	-17022.977	0	21377.023
	2	85836	-42094.627	0	43741.373		2	132164	-58589.187	-55698.523	17876.291
	3	8	-3.923	0	4.077		3	87600	-38833.667	0	48766.333
8	1	30200	0	0	30200	29	1	80000	-54291.226	0	25708.774
	2	58524	0	0	58524		2	104070	-70626.099	-11175.873	22268.028
	3	8	0	0	8		3	43800	-29724.446	0	14075.554
9	1	121200	-73147.854	0	48052.146	30	1	62000	-39516.623	0	22483.377
	2	76640	-46254.55	0	30385.45		2	69477.5	-44282.519	-5278.701	19916.28
	3	58	-35.005	0	22.995		3	65700	-41874.873	0	23825.127
10	1	54000	-7440.741	0	46559.259	31	1	358800	-306266.21	0	52533.79
	2	34713	-4783.156	0	29929.844		2	991774	-846563.17	-106058.26	39152.578
	3	4	-0.551	0	3.449		3	136400	-116428.96	0	19971.04
11	1	63200	-7667.753	0	55532.247	32	1	57000	-20777.239	0	36222.761
	2	23931	-2903.434	0	21027.566		2	22890.13	-8343.749	0	14546.381
	3	66	-8.007	0	57.993		3	65700	-23948.502	0	41751.498
12	1	124200	-41109.159	-29799.373	53291.467	33	1	103600	-75161.439	0	28438.561
	2	52744	-17457.822	0	35286.178		2	97113	-70455.143	-4126.644	22531.212
	3	9	-2.979	0	6.021		3	131400	-95330.242	0	36069.758
13	1	166200	-91860.704	0	74339.296	34	1	38800	-12288.747	0	26511.253
	2	130704	-72241.646	-2827.862	55634.492		2	19722	-6246.357	0	13475.643
	3	8	-4.422	0	3.578		3	175200	-55489.392	-35566.973	84143.635
14	1	116400	-54380.678	0	62019.322	35	1	99400	-62784.633	0	36615.367
	2	112874	-52733.373	-11732.373	48408.254		2	40092	-25323.556	0	14768.444
	3	15	-7.008	0	7.992		3	131400	-82996.989	0	48403.011
15	1	129600	-84773.579	0	44826.421	36	1	146000	0	0	146000
	2	92392	-60435.189	0	31956.811		2	174570	0	0	174570
	3	72	-47.096	0	24.904		3	1	0	0	1
16	1	50000	-23696.629	0	26303.371	37	1	208000	-141463.47	-10761.718	55774.808
	2	24457	-11590.969	0	12866.031		2	57020	-38780.035	0	18239.965
	3	219000	-103791.24	-30797.206	84411.559		3	87400	-59441.864	0	27958.136
17	1	80000	-187.589	-29362.196	50450.214	38	1	159400	-121784.85	0	37615.155
	2	35886	-84.148	0	35801.852		2	88655	-67734.225	0	20920.775
	3	2	-0.005	0	1.995		3	182500	-139433.72	0	43066.284
18	1	94000	-72213.266	0	21786.734	39	1	271200	-177740.03	-29925.994	63533.977
	2	476302	-365907.69	-92403.768	17990.541		2	133403	-87430.137	0	45972.863
	3	219000	-168241.55	0	50758.455		3	6	-3.932	0	2.068
19	1	144400	-114101.79	0	30298.206	40	1	44000	-11270.573	0	32729.427
	2	141100	-111494.21	-4854.555	24751.24		2	16728	-4284.867	0	12443.133
	3	73000	-57683.04	0	15316.96		3	73000	-18698.905	0	54301.095
20	1	237000	-207472.98	0	29527.023	41	1	45800	0	0	45800
	2	170235	-149026	0	21208.999		2	7300	0	0	7300
	3	109500	-95857.768	0	13642.232		3	109500	0	0	109500
21	1	244800	-213136.98	0	31663.025	42	1	57800	-33399.308	0	24400.692
	2	313044	-272554.13	-15419.728	25070.141		2	87120	-50341.656	-16223.275	20555.069
	3	178080	-155046.7	0	23033.299		3	74200	-42875.928	0	31324.072

**Table 7.** Projection summary.

Farm	Input	Original movement	Radial movement	Slack Value	Projected	Farm	Input	Original movement	Radial movement	Slack value	Projected
43	1	40600	-17261.07	0	23338.93	64	1	168000	-129510.36	0	38489.638
	2	30680	-13043.587	0	17636.413		2	89297	-68838.612	0	20458.388
	3	80300	-34139.506	0	46160.494		3	153300	-118178.21	0	35121.795
44	1	70000	-47098.422	0	22901.578	65	1	158000	-115496.56	0	42503.438
	2	144105	-96958.83	-27886.97	19259.2		2	207716	-151838.51	-26150.543	29726.952
	3	120450	-81042.928	0	39407.072		3	175200	-128069.61	0	47130.395
45	1	93600	-52513.743	-886.178	40200.079	66	1	129800	-102912.68	0	26887.324
	2	28180	-15810.227	0	12369.773		2	114758	-90986.54	-1598.098	22173.362
	3	131400	-73721.217	0	57678.783		3	131400	-104181.25	0	27218.755
46	1	167600	-94959.588	-14428.504	58211.908	67	1	44000	-26731.077	0	17268.923
	2	94200	-53372.274	0	40827.726		2	49180	-29878.054	-4853.737	14448.209
	3	8	-4.533	0	3.467		3	175200	-106438.29	0	68761.712
47	1	142800	-114454.07	0	28345.929	68	1	196800	-169165.04	0	27634.963
	2	178562	-143117.28	-12560.257	22884.461		2	139496	-119907.75	0	19588.246
	3	146000	-117018.87	0	28981.131		3	153300	-131773.38	0	21526.625
48	1	68800	-44262.961	0	24537.039	69	1	189000	-136509.22	0	52490.778
	2	69320	-44597.507	-4086.854	20635.64		2	68960	-49807.809	0	19152.191
	3	87600	-56358.073	0	31241.927		3	153300	-110724.15	0	42575.853
49	1	50000	-20258.526	0	29741.474	70	1	207400	-170328.59	0	37071.406
	2	117160	-47469.779	-48341.602	21348.619		2	145900	-119821.32	0	26078.679
	3	116800	-47323.917	0	69476.083		3	153300	-125898.62	0	27401.381
50	1	94000	-69038.531	0	24961.469	71	1	98000	-38049.179	-10921.489	49029.331
	2	147260	-108155.47	-17444.891	21659.641		2	55336	-21484.586	0	33851.414
	3	65700	-48253.526	0	17446.474		3	8	-3.106	0	4.894
51	1	905200	-377259.57	-133252.58	394687.857	72	1	137600	-108162.29	0	29437.715
	2	533824	-222481.45	0	311342.547		2	93685	-73642.323	0	20042.677
	3	5	-2.084	0	2.916		3	255500	-200839.13	0	54660.874
52	1	174400	-35928.028	0	138471.972	73	1	77800	-29378.9	0	48421.1
	2	207792	-42807.092	-58120.202	106864.706		2	49350	-18635.587	-360.511	30353.901
	3	4	-0.824	0	3.176		3	153300	-57889.272	-1588.966	93821.762
53	1	38826000	0	0	38826000	74	1	151000	-114916.4	0	36083.602
	2	44020690	0	0	44020690		2	90696	-69022.898	0	21673.102
	3	2701000	0	0	2701000		3	204400	-155555.71	0	48844.293
54	1	11275000	-2685871	0	8589128.99	75	1	220600	0	0	220600
	2	14580794	-3473359.8	-4227887	6879547.23		2	119860	0	0	119860
	3	1591400	-379094.91	-1171022.9	41282.237		3	204400	0	0	204400
55	1	10950000	-2279099.7	-711716.3	7959183.95	76	1	69800	-6926.584	-17910.113	44963.304
	2	11490914	-2391683.9	-2698610.1	6400619.95		2	35587	-3531.466	0	32055.534
	3	5	-1.041	0	3.959		3	5	-0.496	0	4.504
56	1	10706800	0	0	10706800	77	1	76600	-23667.192	-440.234	52492.574
	2	8590098	0	0	8590098		2	51825	-16012.431	0	35812.569
	3	5	0	0	5		3	6	-1.854	0	4.146
57	1	12198000	-2005493.3	0	10192506.7	78	1	126000	-97998.736	0	28001.264
	2	14671234	-2412121.8	-4084435.1	8174677.09		2	100980	-78538.987	0	22441.013
	3	1898000	-312053.31	-1575917.2	10029.507		3	102200	-79487.863	0	22712.137
58	1	102500	-41591.214	0	60908.786	79	1	93600	-67252.192	0	26347.808
	2	84589	-34323.504	0	50265.496		2	69840	-50180.482	0	19659.518
	3	75	-30.433	0	44.567		3	131400	-94411.731	0	36988.269
59	1	44000	-17902.953	0	26097.047	80	1	4174000	-3599624.4	-430089.93	144285.646
	2	30895	-12570.721	0	18324.279		2	593648	-511957.32	0	81690.683
	3	67160	-27326.417	0	39833.583		3	177750	-153290.19	0	24459.813
60	1	279400	-221210	0	58189.996	81	1	248800	-210777.38	0	38022.617
	2	128975	-102113.67	0	26861.327		2	149189	-126389.34	0	22799.663
	3	153300	-121372.56	0	31927.439		3	350400	-296850.46	0	53549.537
61	1	203000	-125800.17	-29069.4	48130.429	82	1	247800	-209024.91	0	38775.095
	2	40336	-24996.432	0	15339.568		2	226043	-190672.38	-7636.382	27734.235
	3	205800	-127535.35	0	78264.654		3	292000	-246308.61	0	45691.395
62	1	95400	-73140.314	0	22259.686	83	1	183600	-149925.71	0	33674.288
	2	94813	-72690.279	-3013.02	19109.701		2	157408	-128537.62	-4041.664	24828.72
	3	153300	-117530.51	0	35769.495		3	255500	-208638.45	0	46861.55
63	1	5202000	-1041708.2	-3291390.9	868900.876	84	1	38000	-7370.988	0	30629.012
	2	804660	-161134.35	0	643525.648		2	13212	-2562.776	0	10649.224
	3	562100	-112561.35	-257775.2	191763.444		3	219000	-42480.169	-85756.098	90763.733

**Table 8.** Projection summary.

Farm	Input	Original movement	Radial movement	Slack value	Projected
85	1	262200	-224975.98	0	37224.024
	2	180574	-154938.26	0	25635.74
	3	182500	-156590.83	0	25909.17
86	1	63600	0	0	63600
	2	20145	0	0	20145
	3	44	0	0	44
87	1	75600	-18112.5	-11687.5	45800
	2	9600	-2300	0	7300
	3	365000	-87447.917	-168052.08	109500
88	1	56000	-10313.547	0	45686.453
	2	50049	-9217.548	-7182.118	33649.333
	3	5	-0.921	0	4.079
89	1	129600	-51316.294	0	78283.706
	2	95901	-37972.87	0	57928.13
	3	4	-1.584	0	2.416
90	1	16000	0	0	16000
	2	13500	0	0	13500
	3	73000	0	0	73000
91	1	50000	0	0	50000
	2	35148	0	0	35148
	3	2	0	0	2
92	1	45600	0	0	45600
	2	25111	0	0	25111
	3	4	0	0	4
93	1	36000	0	0	36000
	2	10200	0	0	10200
	3	58400	0	0	58400
94	1	48000	-19636.631	0	28363.369
	2	28147	-11514.839	0	16632.161
	3	58400	-23891.235	0	34508.765
95	1	117200	-59881.796	0	57318.204
	2	49687	-25386.918	0	24300.082
	3	95	-48.539	0	46.461
96	1	50000	-13629.522	0	36370.478
	2	13340	-3636.357	0	9703.643
	3	116800	-31838.564	0	84961.436
97	1	36000	-6258.178	0	29741.822
	2	12880	-2239.037	0	10640.963
	3	116800	-20304.31	-6664.264	89831.426
98	1	77600	-48070.612	0	29529.388
	2	38653	-23944.244	0	14708.756
	3	116800	-72353.704	0	44446.296
99	1	132200	-103752.47	0	28447.535
	2	78113	-61304.208	0	16808.792
	3	175200	-137499.49	0	37700.515
100	1	52000	-8426.796	0	43573.204
	2	44396	-7194.539	0	37201.461
	3	5	-0.81	0	4.19
101	1	103600	-12985.226	0	90614.774
	2	135337	-16963.143	-34707.261	83666.596
	3	2	-0.251	0	1.749
102	1	67600	-32823.657	0	34776.343
	2	41951	-20369.604	0	21581.396
	3	56	-27.191	0	28.809

### Input projected

Tables 6, 7 and 8 display the value of the projected cost to be reduced considering the excess costs assumed for each input, estimated by the multi-stage DEA method. ^49^^,^^50^ It identifies efficient projected points, which have inputs, which is as similar as possible to those of the inefficient point, and invariant to units of measurement. Ferrer and Lovell ^51^^–^^57^ argue that slacks may essentially be viewed as allocative inefficiency. Farms that have negative values are inefficient because they have slack to reduce, that is, they have to reduce their costs (slack) to obtain an optimal level of production compared to farms that are referents or peers. The cost minimization model (VRS) offers a peer evaluation in which each farm has the objective of evaluating the level of costs that need to be reduced to reach the optimum production referenced by the farms indicated as peers. ^9^^,^^58^^–^^60^ These results are relevant to improve production processes in the studied regions. ^13^ It was very important to identify the producers with the best income and therefore with the lowest costs. In the same way, it contributed to identify the necessary cost to reduce in each farm to obtain the optimal conditions of productivity and technical efficiency.

## Conclusions

This study used DEA to investigate the efficiencies of Tlaxcala’s dairy farm for data from 102 farmers in 2020. Using the VRS model and multi-stage method the efficiency of the Tlaxcala dairy farm was assessed.

In conclusion, the CRS, VRS model was used to measure the technical efficiency and productivity of the farms under study, managing to measure both their efficiency and their level of slack. A second conclusion was that 11 farms stood out for their optimal levels of production and their reduced costs, in such a way that in the CRS model, VRS marks the benchmarks as pairs, demonstrating that 50% of the farms were below this condition. optimization or were in scale of deficient constant returns. A third conclusion is that the farms that had to reduce their costs to be located at the optimum level of production were identified. A fourth conclusion was that it was quantified through cost reduction projections for farms that it was necessary to make adjustments in their costs.

We have put together some recommendations based on our findings that would provide Tlaxcala Mexico with policy directives to minimize cost streams. In the first place, the input cost of investment in livestock must be reduced, or the quality of the cattle herd should be improved by investing in genetic improvement of the inventory that is held. The second input referred to the total annual cost for fuel, feeding, reproduction, illness and treatment, milking, mortality, and preventive medicine, based on the results found, it suggests improving good livestock management practices, and for input 3 referred to the annual cost of family and hired labor was suggested reviewing the investment of the time in the face of technical efficiency, pharmaceutical, sanitation.

## Ethics statement

The protocol to carry out this research was reviewed and confirmed to proceed by the Colegio de Postgraduados (Institución de Enseñanza e Investigación en Ciencias Agrícolas). No formal ethical approval was required for this study as per the ‘Ley General de Protección de Datos Personales en Posesión de Sujeto Obligados’, regarding ethical approval requirements for this type of study. The questionnaire included a verbal statement requesting the consent of the producers in accordance with the provisions of the general law on the protection of personal data held by obligated subjects. Verbal as opposed to written consent was used because the aforementioned law does not require written consent to be bound by its compliance.

## Author contributions

Conceptualization: Carlos Zuniga

Methodology: Carlos Zuniga

Formal analysis: Carlos Zuniga, Jose Luis Jaramillo, Noel E. Blanco Roa

Investigation: Carlos Zuniga, Jose Luis Jaramillo, Noel E. Blanco Roa

Writing - original draft: Carlos Zuniga

Validation: Carlos Zuniga, Jose Luis Jaramillo, Noel E. Blanco Roa

Writing – review & editing: Carlos Zuniga, Jose Luis Jaramillo, Noel E. Blanco Roa

Data: Carlos Zuniga & Jose Luis Jaramillo

## Data Availability

Figshare: Data for: Inputs-Oriented VRS DEA in dairy farms, https://doi.org/10.6084/m9.figshare.21836133.v4. ^33^ This project contains the following underlying data:
- DataforDEAF1000R.cvs - S3.csv (Dataset used for this study) Figshare: Data for: Inputs-Oriented VRS DEA in dairy farms, https://doi.org/10.6084/m9.figshare.21836133.v4. ^33^ This project contains the following extended data:
- Questionnaire MilkProd.pdf (Questionnaire/interview guide translated to English) - Questionnaire de campo_leche.pdf (Questionnaire/interview guide in Spanish) Data are available under the terms of the Creative Commons Attribution 4.0 International license (CC-BY 4.0).
