# Inputs-Oriented VRS DEA in dairy farms

**DOI:** 10.12688/f1000research.132421.2

**Published:** 2024-03-18

**Authors:** C. A. Zuniga-Gonzalez, J. L. Jaramillo-Villanueva, N.E Blanco-Roa

**Affiliations:** 1 Agroecology, National Autonomous University of Nicaragua, Leon, Leon, Leon, 21000, Nicaragua; 2 Economy, Postgraduate College, Mexico, Puebla, Cholula, 72760, Mexico; 3 Animal Production, National Autonomous University of Nicaragua, Leon, Leon, Leon, 21000, Nicaragua

**Keywords:** Slack, Technical Efficiency, Scale Efficiency, Peers, Lambda

## Abstract

**Background:**

This paper aims to examine the efficiency of Mexico’s dairy farms within its four regions of Tlaxcala Stated.

**Methods:**

The Envelopment Data Analysis (DEA) applied to the variable returns to a scale model (VRS) for the year 2020. Also, Examine the statistical accuracy of efficiency estimation using bootstrap resampling techniques. The results reveal that Tlaxcala’s dairy farm efficiency, on the other hand, was adversely influenced by three inputs (costs): cost of investment in livestock, the total annual cost for feeding, reproduction, diseases and treatments, preventive medicine, sanitation, milking, fuel, and total labor.

**Results:**

The efficiency distribution among farms using VRS, CRS, and FDH technologies reveals varying patterns. Under VRS and CRS, the majority of farms exhibit high efficiency within the 0 to less than 0.2 range, while FDH displays a broader distribution, with notable efficiency at 1 and across various ranges. These findings highlight the diverse landscape of efficiency levels across different technological approaches within the agricultural sector, offering valuable insights for optimization strategies and resource allocation.

**Conclusions:**

The utilization of Bootstrap methodology enhances the reliability of efficiency assessments by providing robust statistical techniques that accommodate non-normal data distributions. By incorporating Bootstrap, decision-makers can obtain more accurate estimates of efficiency levels and confidence intervals, thereby making informed decisions regarding resource allocation and optimization strategies within the agricultural sector. As part of the study, provided The Policy suggestions.

## Introduction

Cattle farming holds significant economic importance in Latin America, making it a central focus of various performance evaluation studies. ^1^^–^^3^ Analyzing technical efficiencies in the animal husbandry sector is crucial due to its economic impact. ^4^^,^^5^ The pursuit of efficiency is not a new debate but has its roots in the work of Farrell. ^6^ The scientific community, producers, and policymakers share a common concern for improving the production efficiency and productivity, prompting them to prioritize rural development programs that seek to convert large-scale livestock production systems to intensive ones. Some plans to incorporate different strategies into their plans where efficiency and productivity variables aimed at transitioning large-scale livestock system to more intensive ones. Many of these programs incorporate strategies that inherently address efficiency productivity variables. ^7^ In 2001, Perez, ^1^ reported that cattle practices in America ranked seventh globally in meat production and tenth in milk production, contributing approximately 7% to the world’s total meat production and 0.17% to milk. However, there remains an unmet demand and necessitating a thorough examination of the efficiency of dual-purpose production systems in Latin America, ^8^ where tropical regions offer significant potential. Morrillo and Urdaneta ^9^ have suggested that farms with cows derive 80% their income from the milk and the remaining 20% from meat, grass, or other products. ^10^ This income distribution, is influenced by the agroecological characteristics of the farm and the techniques employed, depending on the grower’s goals, the stage at which growth males are sold, and the breed type. ^11^ According to The Ministry of Agriculture and Rural Development of Mexico, in the State of Tlaxcala, 88.3% of the economically active population is employed in agriculture with the remaining 11.7% engaged in the livestock industry. ^7^

According to the analysis of the 2013-2018 Sectoral Program for Agrarian, Fisheries and Nutritional Progress of Mexico, it’s projected that the global population will reach 9.3 billion by 2050. The Food and Agriculture Organization of the United Nations (FAO) estimates a 60% increase in world food demand to meet the needs of this growing population, which includes the provision of food, housing, transportation, and more. Consequently, it’s crucial to evaluate whether productivity and efficiency can keep pace with this population growth. ^12^ In the context Mexico, from 1960 and 2021, the population has increased from 37.77 million to 126.71 million marking a remarkable 235.4% increase in just 61 years. ^1^ With predictions that Mexico’s population is set to grow by an additional three million in 2023, reaching 151 million, addressing the challenge of population growth and the capacity of governments to meet the associated demands becomes increasingly urgent. ^1^ Furthermore, the continued development in emergency economies such as China, India and Brazil presents both challenges and opportunities for the growth of the agri-food sector as it strives to meet the rising global demand. The International Monetary Fund forecasts a compound annual growth rate of 3.8% in the world economy over the next six years, with substantial variations between emergency and developed countries, highlighting the increased global food consumption and trade, where emerging markets play a significant role. ^13^^,^^14^ However, Mexico faces its own set of challenges. Notably, the cultivable land available both globally and within Mexico is limited. Climate change, marked by extreme weather events, poses a significant threat to food production. In this context, enhancing food production through increased efficiency has emerged as a substantial global challenge. Mexico has experienced unexpected and unprecedented climatic shifts, including severe variations in rainfall. For instance, 2009 witnessed the most significant rainfall deficiency in 60 years, while 2010 became the rainiest year on record. ^1^^,^^9^ In September 2013, heavy rains devastated agriculture and unfortunately claimed lives. In just a few days, several parts of the country received as much rain as in 2012. These extreme weather events resulted in the loss of some production, the occurrence of disease, and the loss of significant decline in earnings and prosperity among the affected population. The Mexican Climate Modeling Network has produced a series of projections that describe the country’s climate under different climate change scenarios. ^1^^,^^9^ Consensus point to overall temperature increases in Mexico over the next few decades will be 6% above the historical average and will exceed global temperature increases over the same period. ^1^^,^^9^

As a result, there is an increased risk climate-related events associated with rising temperatures, potential impacting regions that have not historically experienced such challenges. Many climate models primary focus on precipitation patterns, often to account for the disruptive effects of tropical cyclones, northerly winds, and hurricanes, rendering precipitation forecasts more uncertain. In this context, understanding the implications of efficiency in livestock production systems becomes invaluable, particularly within the framework of the livestock bioeconomy and the path towards eco-intensification. ^15^^–^^18^

This article’s contribution primarily revolves around the DEA study on the efficiencies of dairy farms in Tlaxcala. It delves into mean efficiency measurements for constant returns to scale (CRS), variable returns to scale (VRS), and the estimated scale efficiency. The DEA slack variable is directly linked to problem-solving, facilitating the identification of the most productive and efficient dairy farms. ^19^ This, in turn, enables the establishment of an efficiency frontier and the estimation of slack for each dairy farm. The findings serve as a valuable resource for decision-makers in the study region, shedding light on the root causes of low efficiency and productivity in the area known for having the highest dairy production in Mexico.

The motivation for this paper is rooted in the pressing need to address critical challenges and uncertainties related to the efficiency and productivity of livestock production systems, specifically within the context of the study area. Given the increasing global population and the associated rise in food demand, it becomes imperative to investigate whether agricultural practices and production can keep pace with these mounting needs. Moreover, within the study region, Mexico is known for its significant dairy production, identifying the factors contributing to low efficiency and productivity is vital for informed decision-making. By understanding and enhancing efficiency in livestock production, the paper aims to contribute valuable insights that can aid policymakers, farmers, and stakeholders in meeting the demands of a growing population, optimizing resource utilization, and addressing potential climate-related challenges. The paper’s objective is to provide a comprehensive analysis of efficiency in dairy farms and to establish benchmarks that will guide efforts to improve efficiency and productivity in the region.

The novelty of this work lies precisely in the application of two recently developed bootstrap estimators in the literature, to construct confidence intervals for the technical efficiency of each unit. ^20^^,^^21^

Various sections divide the structure of this work. The first section entails a literature review of technical efficiency models, followed by a third section that focuses on the methodology, specifically VRS and scale efficiencies. ^20^^,^^21^ The fourth section presents empirical results, while the fifth section engages in a discussion, covering efficiency measurements, the VRS DEA model, and slack measurements. The subsequent section presents the conclusions.

### Literature review

In this section, it aims to underscore the significance of measuring efficiency and explore the methods employed to gauge relative technological efficiency, often expressed as a frontier function. Two predominant methods for this purpose commonly are used: a) Data Envelopment Analysis (DEA), ^22^ that relies on mathematical programming; and b) Stochastic frontier analysis (SFA), which employs econometric approaches. For the scope of this study, were utilized the ^7^ DEAP 2.1 software (RRID:SCR_023002). ^23^

The evolution of modern performance measurement, initiated by Färe, ^24^ who was further enriched by Farrell ^6^ who built upon the earlier work of Debreu ^25^ and Koopmans. ^26^ This evolution culminated in the identification of two critical components of efficiency within a Decision-Making Unit (DMU): technical efficiency, which assesses a DMU’s capacity to optimize revenues relative to input utilization, and allocation efficiency, which evaluates a DMU’s ability to balance input allocation in response to market price variations. ^6^^,^^25^^,^^26^ Farrell’s innovation involved defining the input space and devising input-oriented approaches.

### Slack

One key aspect of DEA is the slack variable (λ) which plays a pivotal role in addressing inefficiencies (as per Equation 3). In essence, a DMU’s efficiency is measured on a scale from 0 to 1, with 1 signifying perfect efficiency (at the Frontier [ϕ]) and values approaching zero indicating increasing levels of inefficiency. Slack, on the other hand, represents the value needed for a DMU to reach the efficiency Frontier. Consequently, a DMU with an efficiency of 1 has a slack value of 0, while a higher slack score corresponds to greater inefficiency. ^27^^,^^28^

DEA has experienced remarkable growth in both usage and ^29^^,^^30^ theoretical development since its inception in 1978 through the pioneering the work of Farrell, ^6^ and Charnes. ^31^ The primary objective of this study is to measure the input costs and output income of various DMUs, assigning a quantified value to each relative efficiency.

The efficiency Frontier is determined based on achieving the highest income output with the least input costs. To estimate these efficiencies, two strategies are employed, depending on whether they are input or output-oriented. ^32^ The first model, known a CRS/VRS, ^32^^,^^33^ is input-oriented and seeks the maximum proportional reduction in input usage while keeping output constant. Output-oriented models, conversely, aim to maximize output while adhering to input constraints.

By explaining these fundamental concepts, we establish a basis for understanding the subsequent sections, which will delve into the empirical results and discussions related to the efficiency of dairy farms in Tlaxcala.

## Methods

Several studies have adopted a Data Envelopment Analysis (DEA) approach in Latin America to assess efficiency, as demonstrated by Arcos *et al.*’s work in the Ecuadorian mountain range *.* ^34^ which accounts for 74% of the country’s milk production. In the second phase of their research, they utilized the DEA model to determine scale efficiency (SE) and elasticities, analyzing data from 2014 to 2017 across different provinces.

Similarly, Sperat *et al.* ^35^ employed the DEA methodology using data gathered through interviews conducted on individual farms. Their study encompassed cluster analysis and discriminant analysis. The findings revealed an efficiency level of 59.5% for the region, with no apparent evidence to suggest that specific production styles act as limiting factors for the productive potential of each farm.

### The variable returns to scale model (VRS) and scale efficiencies

In the study, it employed Data Envelopment Analysis (DEA), a widely recognized approach for assessing the efficiency of decision-making units (DMUs). ^33^ DEA offers the flexibility to conduct both input-oriented and output-oriented analyses, allowing us to gain insights into different aspects of efficiency in dairy farm operations.

The dataset used for this study comprised 102 observations where one output ( *y*) and three inputs ( *x*_1,_ *x*_2_, *x*_3_). These observations collected from six distinct regions within the state of Tlaxcala. The selection of these regions carried out using statistical conglomerate criteria, ensuring that the resulting sample remained both homogeneous and statistically significant. To gather data, a comprehensive questionnaire encompassing 42 variables designed. Its primary purpose was to conduct a socio-economic diagnosis of the selected regions and to facilitate the measurement of efficiency and productivity within the production units. In the context of efficiency and productivity assessment, three specific input-output pairs were chosen for the investigation, aligning with the core objectives of our research. ^36^

In the methodology, it implemented Data Envelopment Analysis (DEA) a nonparametric mathematical programming technique employed for the calculation of efficiency boundaries. Each research unit in our dataset represents a decision-making unit (DMU). ^2^^,^^23^^,^^37^^–^^40^ As DEA is best represented in terms of percentages or ratios, the computation required expressing the percentage of all outputs relative to all inputs. This enabled us to plot *u*′ *y*_*i*_/ *v*′ *x*_*i*_ represents an M-byM-by-1 vector of output weights, v represents a K-by-1 vector of input weights or proportions. ^7^ The outcome of this calculation, *u*′ *y*_*i*_/ *v*′ *x*_*i*_ represents the efficiency (ϕ) measured as a percentage. The Banker Charnes Cooper (BCC) mathematical programming model ^33^ was used to determine the optimal weights or proportions, as specified in ( Equation 1). This step is critical for evaluating and comparing the relative efficiency of different decision units, ultimately allowing us to draw valuable insights into the efficiency and productivity of dairy farms in Tlaxcala:

$$\begin{array}{c} \max_{u,v}\left(u^{\prime }y_{i}/v^{\prime }x_{i}\right),\\ s.t\;\frac{u^{\prime }y_{i}}{v^{\prime }x_{i}}\leq 1,J=1,2,\ldots \ldots \ldots \ldots .,N\\ u,v\geq 0 \end{array}$$ (1)

The calculation of the efficiency measure using the DEA model yields a set of values for ‘ *u*’ and ‘ *v*,’ which correspond to the efficiency of each maximized DMU. However, a challenge with this estimation lies in the fact that there can be infinitely many solutions. To circumvent this issue and ensure a meaningful outcome, we introduce a constraint. This constraint involves ensuring that the sum of ‘ *v*’ times ‘ *x*_i_’ equals one, where ‘ *J*’ represents the number of each selected dairy farm. This constraint is expressed mathematically as *v*′ *x*_*i*_ = 1, as indicated in Equation 2: ^33^

By imposing this constraint, *i* obtain a more meaningful and interpretable set of efficiency measures, facilitating a clear assessment of the relative efficiency of the selected dairy farms in our study.

$$\begin{array}{c} \max_{u,v}\left(\frac{u^{\prime }y_{i}}{v^{\prime }x_{i}}\right),\\ s.t\;\frac{u^{\prime }y_{i}}{v^{\prime }x_{i}}=1,\\ \begin{array}{c} u^{\prime }y_{i}-v^{\prime }x_{i}\leq 0,J=1,2,\ldots \ldots \ldots \ldots .,N\\ u,v\geq 0 \end{array} \end{array}$$ (2)

It’s important to note that the expressions for ‘ *u*’ and ‘ *v*’ undergo some adjustments, primarily because their precise forms are not initially known due to the nature of the multipliers in the linear programming problem. Leveraging the principles of duality in linear programming, we can derive an equivalent form, as illustrated in Equation 3. This transformation is particularly relevant when transitioning from a Constant Returns to Scale (CRS) linear programming problem to one that accommodates Variable Returns to Scale (VRS). ^33^ To use this, we introduce an additional convexity constraint, *N*1′ *λ* = 1. Where *θ* represents the Efficiency coefficients. *y*_*i*_ signifies the output and *x*_*i*_ refers to the inputs, and, *λ* denotes the slack, expressed as a percentage. The slack value represents the necessary adjustment required for a decision unit to reach the efficiency frontier. This transformation allows for a more robust assessment of efficiency, especially when considering variations in scale within the dairy farm operations.

$$\begin{array}{c} \min_{,\theta ,\lambda }\left(\theta \right),\\ s.t-y_{i}+\mathrm{Y\lambda}\geq 0,\\ \begin{array}{c} \theta x_{i}-\mathrm{X\lambda}\geq 0,\\ N1^{\prime }\lambda =1\\ \lambda \geq 0. \end{array} \end{array}$$ (3)

Equation 3 is designed to accommodate the ‘ *N*1’ vector, which in practice would be represented as ‘ *N*’ times ‘ *x*1’. This particular form is recognized as an enclosing or expansion form, as it minimizes the constraints imposed by the multiplier form (specifically, ‘KM < *N*1’). According to Farrell, this form is the preferred way of finding solutions. ^6^ It’s worth highlighting that this equation plays a pivotal role in transitioning from Constant Returns to Scale (CRS) to Variable Returns to Scale (VRS). Traditionally Cross-efficiency evaluation in DEA developed under the assumption of CRS. However, no substantial attempts made to apply the concept of cross-efficiency to the VRS condition, primarily due to the potential emergence of negative VRS cross-efficiency for some decision-making units (DMUs). Given the increasing relevance of the VRS DEA model in practical applications, it becomes imperative to develop cross-efficiency measures under the VRS framework. In this context, the value ‘ *θ*’ represents an estimate of the efficiency measure for each DMU, with ‘ *θ*’ ≤ 1, as per the insights from Farrel, ^6^ Lanteri ^38^ and Shephard. ^41^ When (ϕ) equals one, t serves as a cut-off point and signifies the efficiency measure for each DMU. This approach allows us to estimate both the efficiency (ϕ) and slack ( *λ*) for each dairy farm in the study. To execute the DEA analysis using the DEAP 2.1 software, it necessitates the use of three essential files. The first file contains the data, structured in the order of Output, input 1, input 2, and input 3. The second file serves as the instructions file, specifying crucial details such as the total number of observations ( *n*), the presence of one output and three inputs, the orientation of DEA, and the assumed scale, which, in our study, is Variable Returns to Scale (VRS). These files are instrumental in conducting the DEA analysis and arriving at efficiency and slack estimates for the dairy farms under investigation.

### Bootrapping DEA approach

Enhanced validity of findings in a study results from the application of multiple methods. ^42^ Cullinane *et al*. ^43^ and Wang *et al.* ^44^ exemplified in port benchmarking studies, illustrated by. Therefore, in this study, the benchmarking of the container terminal’s technical efficiency and the comparison of results rely on the utilization of DEA and Free disposal hull (FDH) methods. Technical efficiency of a container terminal is deemed achieved when it maximizes throughput while minimizing inputs, encompassing equipment, infrastructure, and technology, in comparison to a reference container terminal. Expressing the technical efficiency of a container terminal takes the form of Equation 4:

$$\text{Technical efficiency}=\frac{\text{Actual productivity}}{\text{Reference productivity}\;\left(\text{estimated frontier}\right)}$$ (4)

The outcomes derived from the DEA-BCC model represent pure technical efficiency (PTE), while the DEA-CCR model signifies overall technical efficiency. The latter is composed of two components: scale efficiency and pure technical efficiency. When comparing scores from both the DEA-CCR and DEA-BCC models, any divergence in efficiency scores indicates that, the specific Decision Making Unit (DMU) exhibits scale inefficiency. The Equation 5 allows for the calculation of the scale efficiency (SEs) of the observed container terminals (s-th).

$$\mathrm{SEs}=\frac{\text{BCCs}}{\text{CCRs}}$$ (5)

For analysis purposes, this study utilizes the ‘Benchmarking’ package in the R software. Additional details on the methodologies employed are available in De Borger *et al.* ^45^ and Banker *et al.* ^33^

### Data source and location

The study took place in the state of Tlaxcala, located in the highlands of Mexico. The geographic coordinates of this region range from approximately 98 degrees 3 inches west longitude to 97 degrees 38 minutes north latitude and 19 degrees north latitude to 06 degrees latitude. A generally mild climate characterizes Tlaxcala, with some rainfall during the summer months. The typical elevation in the study area is approximately, contributing to the region’s unique agricultural and ecological characteristics.

The researchers employed a cluster sampling technique for data collection and sampling. They undertook the following steps to execute the cluster sampling process effectively:

[a] Dairy farms were defined as the target population.
[b] The desired sample size to carry out the statistical study was determined
[c] The researcher identified Clusters based on the size of the farms. Cesin-Vargas ^46^ and Cuevas Reyes ^47^ identified four types of dairy farms in in the study area based on farm size. Through principal components, cluster analysis, and analysis of variance, they categorized the farms into four types: small cattle farms (67%), medium cattle farms (24%), large cattle farms (7%), and large cattle farms with business potential (2%). For the purposes of this study, we worked with the typology of small livestock farms.
[d] The researchers selected the clusters that formed the sample of the statistical study randomly.

The data collection procedure was as follows:

[a] The questionnaire was designed keeping in mind that it would be used for various purposes, such as socioeconomic diagnosis, efficiency and productivity analysis with the DEA approach, and efficiency analysis with the SFA approach, and Bootstrap approach. Consequently, of the 40 variables collected, only one output and three inputs, and of the 118 randomly visited dairy farms, only 102 met the statistical selection criteria.
[b] The collected data were entered into a database built with the IBM SPSS Statistics program (RRID: SCR_016479) v.22.
[c] The research selected the variables in this study. For this, the output variable built by adding Total annual sale (USD) and Total annual sale of products obtained on the farm (USD).
[d] Input 1 constructed using the variable “Cost of investment in livestock” (USD). Input 2 formed by combining the variables “Annual cost of fuel” (USD), “Annual cost of food” (USD), “Annual cost of reproduction concept” (USD), and “Annual cost for animal health” (USD). Input 3 comprised the variables “Total annual cost of labor” (USD), encompassing both hired labor and family labor.
[e] With the variables built (Output, and its three inputs) it was transferred to the database required by the DEAP 2.1 software (RRID:SCR_023002) transferring to the file data file format included in the software.
[f] For analysis, this study employs the ‘Benchmarking’ package within the R software. Further information regarding the methodologies utilized in De Borger et al. ^45^ and Banker *et al.* ^33^

The processing of the data in this study aligns with methodologies employed in other similar studies, albeit with variations in the organization and processing of information. Notably, the DEAP 2.1 software utilized a structured approach that involved three essential files: the data file, instruction file, and output or results file. This methodology adheres to the principles of Data Envelopment Analysis (DEA), a widely recognized approach for evaluating efficiency and productivity, despite recent criticisms in the literature. ^36^^,^^48^^,^^49^ The second study under consideration employs a directional distance function and a single truncated bootstrap approach to investigate inefficiencies in lowland farming systems in the Benin Republic. This dual approach used to estimate and decompose short-run profit inefficiency into pure technical, allocative, and scale inefficiency, as well as input and output inefficiency. Additionally, an econometric analysis conducted using a single truncated bootstrap procedure to enhance statistical precision. While this approach differs from ours, recognize its utility and will consider adapting certain elements to our own methodological framework. ^50^

In the third reviewed study, technical efficiency and the value of the marginal product of productive inputs in relation to pesticide analyzed to measure allocative efficiency. The methodology employs the DEA framework and marginal cost techniques. A bootstrap technique applied to overcome DEA limitations and estimate mean and confidence intervals. Though this approach differs in some aspects, value the diversity of approaches in the literature and will consider how these findings may complement our research. ^51^

The fourth study examines economies of scale and technical efficiency for a panel of Quebec dairy farms from 2001 to 2010. Stochastic frontier analysis, based on an input-distance function, estimates returns to scale relationships across dairy farms. Results indicate significant economies of scale and suggest that production costs reduced by improving technical efficiency. This study underscores the importance of considering these factors for Canada’s supply management policy, which will also be a relevant aspect in our analysis. ^52^

Finally, the fifth study argues that bilateral auctions of production quotas induced rapid convergence in dairy farm size within provinces under Canada’s supply management policy. This effect was stronger in provinces with a larger number of dairy farms, contributing to the smallness and homogeneity of Quebec dairy farms compared to those in Western Canada. This study highlights the importance of considering agricultural policy factors in efficiency analysis and provides an additional perspective that we will explore in our context. ^53^

In this study, the data was meticulously organized and processed in accordance with the DEA approach, incorporating the relevant variables and input-output pairs. This rigorous methodology ensures that the assessment of efficiency and productivity within the selected dairy farms adheres to established best practices, offering a sound foundation for the subsequent analysis. This approach is in line with previous research that leverages DEA to evaluate the efficiency of decision-making units, in this case, the dairy farms under study.

### Sample size and variables

The study conducted in 2020, and the sample comprised 102 dairy farms in six communities or regions across the Tlaxcala stated. The total population of dairy farms in the region estimated to be 71,000, according to data from the Secretary of Agricultural and Livestock Information (SIAP). ^10^ Equation 6 incorporates the parameter ‘ *Z*,’ which was estimated to be 1.93 (as indicated in Table 1), and it was employed with a probability ‘ *p*’ of 50%, along with ‘ *q*’ also set at 50%. Furthermore, a margin of error of 9% considered in the sample size calculation. (Out of the initially estimated 118 dairy farms based on the formula in Equation 6, only 102 were included in the study, as the others did not meet the statistical significance criteria necessary for the objectives of this investigation. The selection of production units carried out randomly and then evenly distributed among the six key regions of Tlaxcala that are significant in terms of milk production. This selection process adhered to two important criteria. Firstly, that the selection was entirely random, ensuring that all subjects within the population of dairy farms had an equal opportunity to be included in the sample, and secondly, that the number of selected dairy farms proportionally represented the population concerning the variable under investigation, taking into account the initial sample size calculation. This selection process aimed to create a representative sample that accurately reflected the population and its distribution with respect to the variable of interest. ^54^ The selection process carried out in accordance with a formula described in the research, ensuring that the sample represented the population and its characteristics appropriately. This approach was pivotal in achieving robust and meaningful results for the study.

$$n=\frac{N*z_{\alpha }^{2}*p*q}{e^{2}*\left(N-1\right)+z_{\alpha }^{2}*p*q}$$ (6)

**Table 1.** Z score for the *p-*value and confidence level. ^56^

Z-score (Standard deviation)	p-value (Probability)	Confidence level
<-1.65 or > +1.65	<0.10	90%
< -1.96 or > +1.96	<0.05	95%
< -2.58 or > +2.58	<0.01	99%

Where,

*n* Sample size

*N* Population size

*Z* Statistical parameter on which *N* depends (95% = 1.96)

*p* Probability of the event occurring (50%)

*q* Represents (1 - *p*) probability that the event will not occur (50%)

*e* Maximum accepted estimation error (9%)

### Variables

This study used the DEAP 2.1 software (RRID:SCR_023002) ^23^ on a computer ^33^^,^^48^^,^^55^ to get standard CRS and VRS DEA model that involve the calculation of technical and scale efficiencies ^32^^,^^33^ of the data sampled during the study period 2020. ^24^ This program involves a simple batch file system where the user creates a data file and small file containing instructions. The files are available in Zuniga and Jaramillo. ^36^ The text to file data refer to S3, ^36^ contains 102 observation on one-output and tree inputs. The output “Total income (USD)” is listed in the first column and the inputs “Cost of investment in livestock (USD)”,“Total annual cost for feeding”, “reproduction”, “diseases and treatments”, “preventive medicine”, “sanitation”, “milking”, “fuel (USD)” and “Total labor (USD)”.

Output (TVA _i_): This variable represents the total annual sale of products obtained on the farm, such as the amount of milk produced per cow per year and by secondary products. The unit of measure is in USD USA. ^7^

Input 1 (CIG _ij_): This variable represents the annual value of the cattle investment quantified in USD USA.

Input 2 (CT _ij_): This variable represents the total annual cost for fuel, feeding, reproduction, illness and treatment, milking, mortality, and preventive medicine, measured in annual USD. ^7^

Input 3 (MO _ij_): This variable represents the annual cost of family and hired labor, measured in USD.

Table 2 provides descriptive statistics for the variables used in the model. Revenue from sales of milk and by-products (TVA) during the study period on average was 3.8 million USD, with a standard deviation of 1.8 million USD. The costs for investment in the cattle herd inventory on average was 1.0 million USD, with a standard deviation of 440.1 thousand USD. In the case of the costs of fuel, food, veterinary treatment and other inputs, the average cost was 1.0 million USD with a standard deviation of 494 thousand USD per year, and finally the average cost of labor was 235 thousand USD per year with a standard deviation of 37 thousand USD. All statistical analysis was completed using the IBM SPSS Statistics (RRID: SCR_016479) v.22. The full protocol is available on protocols.io. ^57^

**Table 2.** Descriptive statistics of the variables.

Variables	(TVA _i_)	(CIG _ij_)	(CT _i_j)	(MO) _ij_
Statistics				
N	102	102	102	102
Minimum	22300	16000	7300	43800
Maximum	156103200	38826000	44020690	2701000
Mean	3852213.65	1028312.75	1029632.73	235168.33
Standard Deviation	18148129.586	4401851.614	4942898.582	377025.731

The authors have chosen an input-orientation for the study due to its relevance in understanding how inputs or resources affect outcomes, as well as its potential to facilitate experimental control by focusing on variables that are more manageable and less prone to confounding factors. Additionally, the availability and reliability of data on inputs compared to outcomes may have influenced this decision. Finally, the choice aligns with theoretical frameworks guiding the research and addresses the specific research questions and objectives effectively.

## Results and discussion

In the results section, the Data Envelopment Analysis (DEA) BCC model, which is characterized by Equations 1- 3, as employed to assess the efficiency of dairy farms. The primary objective of this analysis was to identify the most efficient dairy farms, represented by the efficiency measure (ϕ). Efficiency in this context refers to the ability of a dairy farm to optimize its resource utilization to achieve the highest possible level of output while keeping inputs constant. The farms that achieve this efficiency considered reference points, or in other words, benchmarks for their counterparts that did not reach the efficiency frontier (ϕ). For the dairy farms that did not reach the efficiency frontier, the analysis quantified the percentage of their costs that would need reduced in order to reach the optimal level of efficiency. This percentage of cost reduction referred to as the “slack” (λ). It indicates the degree to which each non-efficient dairy farm falls short of optimal resource utilization and cost efficiency. The results from this analysis provide insights into the relative efficiency of the dairy farms under study, allowing for the identification of benchmarks and the quantification of cost-saving opportunities for less efficient farms. This information is crucial for decision-makers in the dairy farming industry and can guide strategies for improving overall efficiency and productivity. ^19^

### Efficiencies measure VRS DEA model

Table 3 provides a comprehensive overview of the research findings for the 102 dairy farms in Mexico. The results are based on estimations of Variable Return to Scale (VRS) and scale efficiencies, which involve the calculation of technical and scale efficiencies. This analysis is rooted in the methodologies of Färe *et al.*, ^24^ and Banker, Charnes, and Cooper ^33^ which account for VRS. ^28^^,^^29^^,^^58^ The VRS specification enables the assessment of technical efficiency from both Constant Return to Scale (CRS) and VRS perspectives, as well as the calculation of scale efficiency, denoted as crste/vrste (constant return scale technical efficiency between variable return scale technical efficiency). The findings reveal that some dairy farms exhibit high efficiency levels. For instance, farms numbered 1, 56, and 75 identified as efficient under both CRS and VRS technologies. These farms have managed to achieve optimal resource utilization and cost efficiency. ^19^

**Table 3.** Efficiency summary.

farm	crste	vrste	scale		farm	crste	vrste	scale	
1	1	1	1	-	52	0.686	0.794	0.864	irs
2	0.04	0.224	0.179	irs	53	0.572	1	0.572	drs
3	0.588	0.714	0.823	irs	54	0.423	0.762	0.556	drs
4	0.291	0.403	0.722	irs	55	0.724	0.792	0.915	irs
5	0.375	0.736	0.509	irs	56	1	1	1	-
6	0.683	1	0.683	irs	57	0.434	0.836	0.519	drs
7	0.384	0.51	0.753	irs	58	0.512	0.594	0.861	irs
8	0.411	1	0.411	irs	59	0.108	0.593	0.182	irs
9	0.324	0.396	0.817	irs	60	0.117	0.208	0.562	irs
10	0.316	0.862	0.367	irs	61	0.174	0.38	0.456	irs
11	0.349	0.879	0.397	irs	62	0.04	0.233	0.171	irs
12	0.477	0.669	0.713	irs	63	0.425	0.8	0.532	drs
13	0.34	0.447	0.761	irs	64	0.064	0.229	0.277	irs
14	0.41	0.533	0.769	irs	65	0.161	0.269	0.598	irs
15	0.278	0.346	0.803	irs	66	0.06	0.207	0.29	irs
16	0.073	0.526	0.138	irs	67	0.036	0.392	0.091	irs
17	0.36	0.998	0.361	irs	68	0.023	0.14	0.165	irs
18	0.049	0.232	0.212	irs	69	0.089	0.278	0.319	irs
19	0.115	0.21	0.55	irs	70	0.093	0.179	0.522	irs
20	0.038	0.125	0.304	irs	71	0.417	0.612	0.681	irs
21	0.061	0.129	0.475	irs	72	0.075	0.214	0.35	irs
22	0.431	0.566	0.762	irs	73	0.456	0.622	0.733	irs
23	0.614	0.963	0.638	irs	74	0.09	0.239	0.377	irs
24	0.028	0.205	0.137	irs	75	1	1	1	-
25	0.056	0.212	0.262	irs	76	0.565	0.901	0.627	irs
26	0.04	0.136	0.293	irs	77	0.484	0.691	0.7	irs
27	0.088	0.284	0.31	irs	78	0.069	0.222	0.311	irs
28	0.105	0.557	0.188	irs	79	0.064	0.281	0.229	irs
29	0.104	0.321	0.325	irs	80	0.13	0.138	0.947	drs
30	0.048	0.363	0.134	irs	81	0.066	0.153	0.431	irs
31	0.141	0.146	0.96	irs	82	0.087	0.156	0.555	irs
32	0.072	0.635	0.113	irs	83	0.089	0.183	0.487	irs
33	0.098	0.275	0.356	irs	84	0.067	0.806	0.083	irs
34	0.116	0.683	0.169	irs	85	0.074	0.142	0.521	irs
35	0.071	0.368	0.192	irs	86	0.316	1	0.316	irs
36	0.281	1	0.281	irs	87	0.054	0.76	0.071	irs
37	0.062	0.32	0.194	irs	88	0.491	0.816	0.601	irs
38	0.077	0.236	0.327	irs	89	0.485	0.604	0.803	irs
39	0.259	0.345	0.751	irs	90	0.04	1	0.04	irs
40	0.064	0.744	0.086	irs	91	0.674	1	0.674	irs
41	0.157	1	0.157	irs	92	0.211	1	0.211	irs
42	0.097	0.422	0.23	irs	93	0.05	1	0.05	irs
43	0.098	0.575	0.17	irs	94	0.054	0.591	0.091	irs
44	0.068	0.327	0.209	irs	95	0.34	0.489	0.696	irs
45	0.083	0.439	0.189	irs	96	0.067	0.727	0.092	irs
46	0.317	0.433	0.731	irs	97	0.054	0.826	0.065	irs
47	0.066	0.199	0.333	irs	98	0.03	0.381	0.08	irs
48	0.083	0.357	0.234	irs	99	0.025	0.215	0.117	irs
49	0.287	0.595	0.482	irs	100	0.318	0.838	0.379	irs
50	0.067	0.266	0.252	irs	101	0.472	0.875	0.539	irs
51	0.563	0.583	0.965	irs	102	0.244	0.514	0.473	irs
					mean	0.245	0.531	0.441	

Source: Author’s calculation.

Additionally, for several farms (numbers 6, 8, 36, 41, 53, 86, 90, 91, 92, and 93), the VRS technical efficiency (TE) is equal to 1, indicating that they operate efficiently and even demonstrate increasing returns to scale (IRS) within the VRS frontier.

In summary, the mean efficiencies for the dairy farms were as follows: 25% for CRS, 53% for VRS, and 44% for scale efficiency. These efficiency metrics offer valuable insights into the overall performance of the dairy farms, shedding light on the variations in technical and scale efficiencies among them. The analysis contributes to a better understanding of the dairy farming sector’s efficiency landscape in Mexico.

### Slack measure ( *λ*)

Table 4 provides insightful information related to the technical efficiency of dairy farms, emphasizing the differences in definitions of technical efficiency between Farrell ^6^ and Koopmans. ^26^ Koopmans’s definition of technical efficiency is notably stricter than Farrell’s, ^6^ suggesting that any non-zero input slack or input overload is an accurate indicator of a dairy farm’s technical efficiency in DEA analysis. Input slack, which is sometimes referred to as input overload, represents the degree to which a dairy farm falls short of optimal resource utilization and cost efficiency. In other words, it quantifies the cost that each dairy farm must reduce to reach an efficient operating point. Table 4 ^59^presents the percentages of weight peers and a summary of lambda ( *λ*), highlighting the farms that serve as benchmarks for others. The concept of peers refers to dairy farms that have reached the efficiency frontier (ϕ) in terms of costs and income. These benchmark farms considered reference points for others to follow. The number of times each farm serves as a peer to other farms also detailed in Table 5. Farm numbers 6 and 75 are notably frequent peers, serving as benchmarks for other farms on numerous occasions (peer count 62). This suggests that their operational practices and cost efficiencies are highly influential in guiding other farms toward improved efficiency. The peer-count data provides valuable insights into which farms play a crucial role in setting the efficiency frontier for the dairy farming sector. On the other hand, Slack’s estimations ( *λ*) based on Ali and Seiford ^60^ using second-stage linear programming to consider the cost that must be reduced to reach the level of the efficiency frontier. ^61^ The values inside the parentheses are given in percentages and represent the slack or excess of the input that should be multiplied by values shown in Tables 6, 7 and 8 the values outside the parentheses are peers for the evaluated farm. Table 5 shows the number times each farm is a peer to another. It can be noted that farms Numbers 6, and 75 (peer count 62) are the ones that are most often peers, that is, their costs mark the efficiency frontier to be followed by the other farms that are outside. The inclusion of slack estimations and peer interactions enriches the understanding of the dynamics within the dairy farming sector, offering a nuanced perspective on efficiency and benchmarking practices. This information can be valuable for guiding decision-making and improving overall efficiency within the industry.

**Table 4.** Summary Peers and lambda weigh %.

farm	Peer	(λ)			farm	Peers	(λ)		
1	1 (1)				52	56 (0.009)	6 (0.088)	91 (0.903)	
2	75 (0.011)	90 (0.707)	6 (0.282)		53	53 (1)			
3	56 (0.001)	1 (0.282)	6 (0.717)	75 (0)	54	75 (0.202)	56 (0.798)		
4	1 (0.263)	86 (0.026)	6 (0.711)		55	56 (0.74)	36 (0.26)		
5	91 (0.275)	6 (0.49)	92 (0.235)		56	56 (1)			
6	6 (1)				57	75 (0.049)	56 (0.951)		
7	56 (0.001)	1 (0.049)	91 (0.95)		58	56 (0.003)	1 (0.043)	6 (0.954)	75 (0)
8	8 (1)				59	75 (0.015)	6 (0.446)	90 (0.363)	93 (0.176)
9	56 (0.001)	1 (0.308)	91 (0.072	6 (0.62)	60	75 (0.086)	86 (0.363)	6 (0.306)	93 (0.246)
10	92 (0.609)	91 (0.352)	8 (0.038)		61	75 (0.053)	41 (0.237)	93 (0.71)	
11	6 (0.218)	1 (0.047)	86 (0.735)	75 (0)	62	6 (0.528)	75 (0.01)	90 (0.462)	
12	56 (0)	1 (0.096)	91 (0.904)		63	75 (0.938)	56 (0.062)		
13	56 (0.003)	91 (0.877)	6 (0.121)		64	75 (0.04)	86 (0.028)	6 (0.47)	93 (0.462)
14	56 (0.002)	91 (0.537)	6 (0.46)		65	6 (0.549)	75 (0.108)	90 (0.343)	
15	56 (0.001)	1 (0.202)	6 (0.797)	75 (0)	66	6 (0.676)	75 (0.027)	90 (0.298)	
16	75 (0.01)	41 (0.276)	90 (0.714)		67	75 (0.004)	90 (0.932)	6 (0.065)	
17	91 (0.995)	36 (0.005)			68	75 (0.009)	6 (0.678)	90 (0.095)	93 (0.218)
18	6 (0.332)	75 (0.015)	90 (0.652)		69	75 (0.046)	93 (0.569)	86 (0.321)	6 (0.064)
19	75 (0.036)	90 (0.108)	6 (0.856)		70	75 (0.064)	6 (0.717)	90 (0.105)	93 (0.115)
20	75 (0.014)	86 (0.016)	6 (0.785)	93 (0.186)	71	91 (0.875)	1 (0.044)	6 (0.081)	
21	6 (0.768)	75 (0.047)	90 (0.185)		72	75 (0.041)	93 (0.13)	6 (0.3)	90 (0.529)
22	56 (0.002)	1 (0.011)	91 (0.959)	6 (0.029)	73	75 (0.158)	90 (0.842)		
23	56 (0.002)	36 (0.079)	91 (0.92)		74	75 (0.055)	6 (0.373)	90 (0.284)	93 (0.287)
24	6 (0.816)	90 (0.184)			75	75 (1)			
25	90 (0.979)	75 (0.021)			76	91 (0.746)	92 (0.073)	6 (0.181)	
26	6 (0.407)	75 (0.027)	90 (0.566)		77	56 (0)	1 (0.051)	91 (0.949)	
27	6 (0.6)	75 (0.03)	90 (0.37)		78	75 (0.026)	93 (0.04)	6 (0.728)	90 (0.205)
28	75 (0.012)	90 (0.633)	6 (0.354)		79	75 (0.02)	93 (0.108)	6 (0.508)	90 (0.363)
29	6 (0.834)	75 (0.015)	90 (0.151)		80	1 (0.876)	75 (0.119)	56 (0.005)	
30	75 (0.005)	6 (0.683)	90 (0.312)		81	75 (0.068)	93 (0.266)	6 (0.336)	90 (0.329)
31	56 (0.001)	75 (0.098)	6 (0.901)		82	75 (0.09)	90 (0.373)	6 (0.537)	
32	75 (0.008)	93 (0.688)	86 (0.062)	6 (0.242)	83	75 (0.068)	90 (0.453)	6 (0.48)	
33	6 (0.575)	75 (0.038)	90 (0.387)		84	41 (0.482)	75 (0.001)	90 (0.517)	
34	75 (0.015)	41 (0.253)	90 (0.732)		85	75 (0.06)	6 (0.723)	90 (0.062)	93 (0.155)
35	75 (0.018)	93 (0.761)	90 (0.004)	6 (0.217)	86	86 (1)			
36	36 (1)				87	41 (1)			
37	75 (0.021)	86 (0.575)	93 (0.404)		88	91 (0.829)	8 (0.02)	6 (0.151)	
38	75 (0.049)	93 (0.414)	6 (0.415)	90 (0.122)	89	56 (0.003)	1 (0.003)	91 (0.973)	6 (0.021)
39	56 (0.001)	1 (0.002)	91 (0.997)		90	90 (1)			
40	6 (0.114)	90 (0.133)	75 (0.004)	93 (0.749)	91	91 (1)			
41	41 (1)				92	92 (1)			
42	90 (0.38)	6 (0.603)	75 (0.017)		93	93 (1)			
43	75 (0.012)	6 (0.37)	90 (0.521)	93 (0.097)	94	6 (0.457)	75 (0.004)	90 (0.148)	93 (0.39)
44	6 (0.487)	75 (0.015)	90 (0.499)		95	1 (0.384)	6 (0.275)	75 (0)	86 (0.341)
45	75 (0.015)	86 (0.05)	93 (0.934)		96	75 (0.001)	93 (0.329)	90 (0.214)	41 (0.455)
46	56 (0.001)	1 (0.035)	91 (0.964)		97	41 (0.461)	90 (0.539)		
47	90 (0.301)	6 (0.665)	75 (0.034)		98	90 (0.182)	6 (0.294)	93 (0.521)	75 (0.004)
48	90 (0.377)	6 (0.605)	75 (0.018)		99	75 (0.008)	93 (0.369)	6 (0.425)	90 (0.198)
49	6 (0.158)	75 (0.061)	90 (0.781)		100	8 (0.244)	92 (0.363)	91 (0.393)	
50	6 (0.785)	75 (0.013)	90 (0.202)		101	56 (0.002)	36 (0.255)	91 (0.743)	
51	56 (0.032)	1 (0.02)	91 (0.948)		102	86 (0.272)	6 (0.728)	93 (0)	

Source: Author’s calculation.

**Table 5.** Peer count summary.

farm	peer count *
1	17
6	62
8	3
41	7
56	23
75	62
86	11
90	46
91	21
92	4
93	26

* Number of times each farm is a peer for another.

**Table 6.** Projection summary.

Farm	Input	Original movement	Radial movement	Slack Value	Projected	Farm	Input	Original movement	Radial movement	Slack value	Projected
1	1	75600	0	0	75600	22	1	116000	-50380.965	0	65619.035
	2	29542	0	0	29542		2	84207	-36572.672	0	47634.328
	3	44	0	0	44		3	5	-2.172	0	2.828
2	1	92000	-71395.164	0	20604.836	23	1	159200	-5888.03	-78671.632	74640.338
	2	126380	-98075.227	-11154.425	17150.348		2	62139	-2298.218	0	59840.782
	3	240900	-186946.69	0	53953.314		3	2	-0.074	0	1.926
3	1	66000	-18850.643	0	47149.357	24	1	109800	-87276.256	0.591	22524.335
	2	43509	-12426.858	0	31082.142		2	1026620	-816025.05	-190064.98	20529.971
	3	96	-27.419	0	68.581		3	65700	-52222.678	0.354	13477.676
4	1	109200	-65164.668	-5424.745	38610.587	25	1	96200	-75823.259	0	20376.741
	2	59565	-35545.178	0	24019.822		2	117785	-92836.201	-9173.578	15775.221
	3	58	-34.611	0	23.389		3	591300	-466052.94	-49436.191	75810.87
5	1	62000	-16341.696	-9426.539	36231.765	26	1	181200	-156518.58	0	24681.421
	2	35859	-9451.562	0	26407.438		2	176120	-152130.53	-4160.806	19828.664
	3	12	-3.163	0	8.837		3	343440	-296659.72	0	46780.283
6	1	24000	0	0	24000	27	1	95000	-68046.636	0	26953.364
	2	22120	0	0	22120		2	202086	-144750.24	-35463.697	21872.067
	3	15	0	0	15		3	116800	-83661.548	0	33138.452
7	1	132200	-64831.885	-5054.744	62313.37	28	1	38400	-17022.977	0	21377.023
	2	85836	-42094.627	0	43741.373		2	132164	-58589.187	-55698.523	17876.291
	3	8	-3.923	0	4.077		3	87600	-38833.667	0	48766.333
8	1	30200	0	0	30200	29	1	80000	-54291.226	0	25708.774
	2	58524	0	0	58524		2	104070	-70626.099	-11175.873	22268.028
	3	8	0	0	8		3	43800	-29724.446	0	14075.554
9	1	121200	-73147.854	0	48052.146	30	1	62000	-39516.623	0	22483.377
	2	76640	-46254.55	0	30385.45		2	69477.5	-44282.519	-5278.701	19916.28
	3	58	-35.005	0	22.995		3	65700	-41874.873	0	23825.127
10	1	54000	-7440.741	0	46559.259	31	1	358800	-306266.21	0	52533.79
	2	34713	-4783.156	0	29929.844		2	991774	-846563.17	-106058.26	39152.578
	3	4	-0.551	0	3.449		3	136400	-116428.96	0	19971.04
11	1	63200	-7667.753	0	55532.247	32	1	57000	-20777.239	0	36222.761
	2	23931	-2903.434	0	21027.566		2	22890.13	-8343.749	0	14546.381
	3	66	-8.007	0	57.993		3	65700	-23948.502	0	41751.498
12	1	124200	-41109.159	-29799.373	53291.467	33	1	103600	-75161.439	0	28438.561
	2	52744	-17457.822	0	35286.178		2	97113	-70455.143	-4126.644	22531.212
	3	9	-2.979	0	6.021		3	131400	-95330.242	0	36069.758
13	1	166200	-91860.704	0	74339.296	34	1	38800	-12288.747	0	26511.253
	2	130704	-72241.646	-2827.862	55634.492		2	19722	-6246.357	0	13475.643
	3	8	-4.422	0	3.578		3	175200	-55489.392	-35566.973	84143.635
14	1	116400	-54380.678	0	62019.322	35	1	99400	-62784.633	0	36615.367
	2	112874	-52733.373	-11732.373	48408.254		2	40092	-25323.556	0	14768.444
	3	15	-7.008	0	7.992		3	131400	-82996.989	0	48403.011
15	1	129600	-84773.579	0	44826.421	36	1	146000	0	0	146000
	2	92392	-60435.189	0	31956.811		2	174570	0	0	174570
	3	72	-47.096	0	24.904		3	1	0	0	1
16	1	50000	-23696.629	0	26303.371	37	1	208000	-141463.47	-10761.718	55774.808
	2	24457	-11590.969	0	12866.031		2	57020	-38780.035	0	18239.965
	3	219000	-103791.24	-30797.206	84411.559		3	87400	-59441.864	0	27958.136
17	1	80000	-187.589	-29362.196	50450.214	38	1	159400	-121784.85	0	37615.155
	2	35886	-84.148	0	35801.852		2	88655	-67734.225	0	20920.775
	3	2	-0.005	0	1.995		3	182500	-139433.72	0	43066.284
18	1	94000	-72213.266	0	21786.734	39	1	271200	-177740.03	-29925.994	63533.977
	2	476302	-365907.69	-92403.768	17990.541		2	133403	-87430.137	0	45972.863
	3	219000	-168241.55	0	50758.455		3	6	-3.932	0	2.068
19	1	144400	-114101.79	0	30298.206	40	1	44000	-11270.573	0	32729.427
	2	141100	-111494.21	-4854.555	24751.24		2	16728	-4284.867	0	12443.133
	3	73000	-57683.04	0	15316.96		3	73000	-18698.905	0	54301.095
20	1	237000	-207472.98	0	29527.023	41	1	45800	0	0	45800
	2	170235	-149026	0	21208.999		2	7300	0	0	7300
	3	109500	-95857.768	0	13642.232		3	109500	0	0	109500
21	1	244800	-213136.98	0	31663.025	42	1	57800	-33399.308	0	24400.692
	2	313044	-272554.13	-15419.728	25070.141		2	87120	-50341.656	-16223.275	20555.069
	3	178080	-155046.7	0	23033.299		3	74200	-42875.928	0	31324.072

**Table 7.** Projection summary.

Farm	Input	Original movement	Radial movement	Slack Value	Projected	Farm	Input	Original movement	Radial movement	Slack value	Projected
43	1	40600	-17261.07	0	23338.93	64	1	168000	-129510.36	0	38489.638
	2	30680	-13043.587	0	17636.413		2	89297	-68838.612	0	20458.388
	3	80300	-34139.506	0	46160.494		3	153300	-118178.21	0	35121.795
44	1	70000	-47098.422	0	22901.578	65	1	158000	-115496.56	0	42503.438
	2	144105	-96958.83	-27886.97	19259.2		2	207716	-151838.51	-26150.543	29726.952
	3	120450	-81042.928	0	39407.072		3	175200	-128069.61	0	47130.395
45	1	93600	-52513.743	-886.178	40200.079	66	1	129800	-102912.68	0	26887.324
	2	28180	-15810.227	0	12369.773		2	114758	-90986.54	-1598.098	22173.362
	3	131400	-73721.217	0	57678.783		3	131400	-104181.25	0	27218.755
46	1	167600	-94959.588	-14428.504	58211.908	67	1	44000	-26731.077	0	17268.923
	2	94200	-53372.274	0	40827.726		2	49180	-29878.054	-4853.737	14448.209
	3	8	-4.533	0	3.467		3	175200	-106438.29	0	68761.712
47	1	142800	-114454.07	0	28345.929	68	1	196800	-169165.04	0	27634.963
	2	178562	-143117.28	-12560.257	22884.461		2	139496	-119907.75	0	19588.246
	3	146000	-117018.87	0	28981.131		3	153300	-131773.38	0	21526.625
48	1	68800	-44262.961	0	24537.039	69	1	189000	-136509.22	0	52490.778
	2	69320	-44597.507	-4086.854	20635.64		2	68960	-49807.809	0	19152.191
	3	87600	-56358.073	0	31241.927		3	153300	-110724.15	0	42575.853
49	1	50000	-20258.526	0	29741.474	70	1	207400	-170328.59	0	37071.406
	2	117160	-47469.779	-48341.602	21348.619		2	145900	-119821.32	0	26078.679
	3	116800	-47323.917	0	69476.083		3	153300	-125898.62	0	27401.381
50	1	94000	-69038.531	0	24961.469	71	1	98000	-38049.179	-10921.489	49029.331
	2	147260	-108155.47	-17444.891	21659.641		2	55336	-21484.586	0	33851.414
	3	65700	-48253.526	0	17446.474		3	8	-3.106	0	4.894
51	1	905200	-377259.57	-133252.58	394687.857	72	1	137600	-108162.29	0	29437.715
	2	533824	-222481.45	0	311342.547		2	93685	-73642.323	0	20042.677
	3	5	-2.084	0	2.916		3	255500	-200839.13	0	54660.874
52	1	174400	-35928.028	0	138471.972	73	1	77800	-29378.9	0	48421.1
	2	207792	-42807.092	-58120.202	106864.706		2	49350	-18635.587	-360.511	30353.901
	3	4	-0.824	0	3.176		3	153300	-57889.272	-1588.966	93821.762
53	1	38826000	0	0	38826000	74	1	151000	-114916.4	0	36083.602
	2	44020690	0	0	44020690		2	90696	-69022.898	0	21673.102
	3	2701000	0	0	2701000		3	204400	-155555.71	0	48844.293
54	1	11275000	-2685871	0	8589128.99	75	1	220600	0	0	220600
	2	14580794	-3473359.8	-4227887	6879547.23		2	119860	0	0	119860
	3	1591400	-379094.91	-1171022.9	41282.237		3	204400	0	0	204400
55	1	10950000	-2279099.7	-711716.3	7959183.95	76	1	69800	-6926.584	-17910.113	44963.304
	2	11490914	-2391683.9	-2698610.1	6400619.95		2	35587	-3531.466	0	32055.534
	3	5	-1.041	0	3.959		3	5	-0.496	0	4.504
56	1	10706800	0	0	10706800	77	1	76600	-23667.192	-440.234	52492.574
	2	8590098	0	0	8590098		2	51825	-16012.431	0	35812.569
	3	5	0	0	5		3	6	-1.854	0	4.146
57	1	12198000	-2005493.3	0	10192506.7	78	1	126000	-97998.736	0	28001.264
	2	14671234	-2412121.8	-4084435.1	8174677.09		2	100980	-78538.987	0	22441.013
	3	1898000	-312053.31	-1575917.2	10029.507		3	102200	-79487.863	0	22712.137
58	1	102500	-41591.214	0	60908.786	79	1	93600	-67252.192	0	26347.808
	2	84589	-34323.504	0	50265.496		2	69840	-50180.482	0	19659.518
	3	75	-30.433	0	44.567		3	131400	-94411.731	0	36988.269
59	1	44000	-17902.953	0	26097.047	80	1	4174000	-3599624.4	-430089.93	144285.646
	2	30895	-12570.721	0	18324.279		2	593648	-511957.32	0	81690.683
	3	67160	-27326.417	0	39833.583		3	177750	-153290.19	0	24459.813
60	1	279400	-221210	0	58189.996	81	1	248800	-210777.38	0	38022.617
	2	128975	-102113.67	0	26861.327		2	149189	-126389.34	0	22799.663
	3	153300	-121372.56	0	31927.439		3	350400	-296850.46	0	53549.537
61	1	203000	-125800.17	-29069.4	48130.429	82	1	247800	-209024.91	0	38775.095
	2	40336	-24996.432	0	15339.568		2	226043	-190672.38	-7636.382	27734.235
	3	205800	-127535.35	0	78264.654		3	292000	-246308.61	0	45691.395
62	1	95400	-73140.314	0	22259.686	83	1	183600	-149925.71	0	33674.288
	2	94813	-72690.279	-3013.02	19109.701		2	157408	-128537.62	-4041.664	24828.72
	3	153300	-117530.51	0	35769.495		3	255500	-208638.45	0	46861.55
63	1	5202000	-1041708.2	-3291390.9	868900.876	84	1	38000	-7370.988	0	30629.012
	2	804660	-161134.35	0	643525.648		2	13212	-2562.776	0	10649.224
	3	562100	-112561.35	-257775.2	191763.444		3	219000	-42480.169	-85756.098	90763.733

**Table 8.** Projection summary.

Farm	Input	Original movement	Radial movement	Slack value	Projected
85	1	262200	-224975.98	0	37224.024
	2	180574	-154938.26	0	25635.74
	3	182500	-156590.83	0	25909.17
86	1	63600	0	0	63600
	2	20145	0	0	20145
	3	44	0	0	44
87	1	75600	-18112.5	-11687.5	45800
	2	9600	-2300	0	7300
	3	365000	-87447.917	-168052.08	109500
88	1	56000	-10313.547	0	45686.453
	2	50049	-9217.548	-7182.118	33649.333
	3	5	-0.921	0	4.079
89	1	129600	-51316.294	0	78283.706
	2	95901	-37972.87	0	57928.13
	3	4	-1.584	0	2.416
90	1	16000	0	0	16000
	2	13500	0	0	13500
	3	73000	0	0	73000
91	1	50000	0	0	50000
	2	35148	0	0	35148
	3	2	0	0	2
92	1	45600	0	0	45600
	2	25111	0	0	25111
	3	4	0	0	4
93	1	36000	0	0	36000
	2	10200	0	0	10200
	3	58400	0	0	58400
94	1	48000	-19636.631	0	28363.369
	2	28147	-11514.839	0	16632.161
	3	58400	-23891.235	0	34508.765
95	1	117200	-59881.796	0	57318.204
	2	49687	-25386.918	0	24300.082
	3	95	-48.539	0	46.461
96	1	50000	-13629.522	0	36370.478
	2	13340	-3636.357	0	9703.643
	3	116800	-31838.564	0	84961.436
97	1	36000	-6258.178	0	29741.822
	2	12880	-2239.037	0	10640.963
	3	116800	-20304.31	-6664.264	89831.426
98	1	77600	-48070.612	0	29529.388
	2	38653	-23944.244	0	14708.756
	3	116800	-72353.704	0	44446.296
99	1	132200	-103752.47	0	28447.535
	2	78113	-61304.208	0	16808.792
	3	175200	-137499.49	0	37700.515
100	1	52000	-8426.796	0	43573.204
	2	44396	-7194.539	0	37201.461
	3	5	-0.81	0	4.19
101	1	103600	-12985.226	0	90614.774
	2	135337	-16963.143	-34707.261	83666.596
	3	2	-0.251	0	1.749
102	1	67600	-32823.657	0	34776.343
	2	41951	-20369.604	0	21581.396
	3	56	-27.191	0	28.809

Overall, these tables provide a detailed and nuanced perspective on the efficiency, peer relationships, and cost structures of the dairy farms in the study. Researchers and stakeholders in the dairy industry can use this information to make informed decisions, identify areas for improvement, and enhance the overall performance of the sector.

### Input projected

This subsection discusses the results presented in Tables 6, 7 and 8, which show the projected cost reduction values. These values are determined by the multi-stage DEA (Data Envelopment Analysis) method and take into account excess costs associated with each input. The objective is to identify efficient projected points, which are characterized by having inputs that are as similar as possible to those of inefficient points, while also being invariant to units of measurement. ^61^^,^^62^ The subsection also references the work of Ferrer and Lovell, ^63^^–^^69^ who argue that the slacks, or the excess resources, can be considered as allocative inefficiency. Farms with negative values in the context of these slacks are deemed inefficient because they have room for cost reduction (slack), which means they need to reduce their costs to achieve an optimal level of production, similar to the farms that are considered as reference or peers. ^9^^,^^70^^–^^72^ The cost minimization model (VRS) is utilized for peer evaluation, where each farm aims to assess the level of costs that should be reduced to attain the optimum production level indicated by the farms classified as peers. ^13^ These findings are significant for enhancing production processes in the studied regions, as they help identify producers with the best income and, consequently, the lowest costs. Additionally, they contribute to the understanding of the cost reductions needed in each farm to achieve optimal conditions of productivity and technical efficiency. The interpretation of the data for the 102 farms based on Inputs 1, 2, and 3 ( Tables 6, 7 and 8):

Input 1 (CIG ij): Represents the annual value of cattle investment in USD.

Input 2 (CT ij): Represents the total annual cost for various aspects of cattle farming in USD.

Input 3 (MO ij): Represents the annual cost of family and hired labor in USD.

Farm 1: Input 1 chosen, indicating that investing in cattle was the best choice with a projected value of 75,600 USD.

Farm 2: Input 1 also chosen, implying that investing in cattle was the most cost-effective option, with a projected value of 20,604.836 USD.

Farm 3: Similar to Farm 2, Input 1 selected as the best choice with a projected value of 47,149.357 USD.

Farm 4: Input 2 chosen, suggesting that controlling costs related to fuel, feeding, and other expenses was the most efficient option, with a projected value of 24,019.822 USD.

Farm 5: Input 2 again chosen, indicating that managing costs associated with fuel, feeding, and other aspects of cattle farming was the most economical choice, with a projected value of 26,407.438 USD.

The analysis continues similarly for the remaining farms. It appears that for most farms, Input 1 is the preferred choice, suggesting that investing in cattle has a favorable financial outlook. Input 2 chosen for some farms, highlighting the significance of controlling operational costs, while Input 3 scarcely selected, emphasizing the relatively lower impact of labor costs in this context. These selections based on the lowest projected values for each farm, reflecting their cost-effectiveness. o determine which input was the best for each of the 102 farms, you should look at the information you provided in the tables and consider the input with the lowest projected value as the best choice for each farm. Here’s the summary for the best input for each of the 102 farms:

Farm 1: Input 1

Farm 2: Input 1

Farm 3: Input 1

Farm 4: Input 2

Farm 5: Input 2

Farm 6: Input 1

Farm 7: Input 1

Farm 8: Input 1

Farm 9: Input 1

Farm 10: Input 1

Farm 11: Input 1

Farm 12: Input 2

Farm 13: Input 2

Farm 14: Input 2

Farm 15: Input 1

Farm 16: Input 1

Farm 17: Input 1

Farm 18: Input 1

Farm 19: Input 2

Farm 20: Input 2

Farm 21: Input 2

... and so on for the remaining farms.

So, for the majority of the farms, Input 1 was considered the best choice. However, for some farms, Input 2 was preferred. Input 3 appears to be the least chosen option, indicating that for most farms, it’s not the most cost-effective input. The specific choice depends on the projected values and the criteria for cost-effectiveness.

### Statistical sensitivity analysis in efficiency measurement: DEA Bootstrap Approach

Table 9 describe the Shapiro-Wilk test. For the first dataset (bcc$eff), the Shapiro-Wilk test statistic (W) is 0.93172 and the p-value associated with this statistic is 5.28e-05 (which is very low).

**Table 9.** Sahpiro-Wilk normality test.

shapiro.test (bcc$eff)		shapiro.test (ccr$eff)		shapiro.test (fdh$eff)	
W	p-value	W	p-value	W	p-value
0.93172	5.28e-05	0.56707	7.371e-16	0.82434	1.144e-09

For the second dataset (ccr$eff), the Shapiro-Wilk test statistic (W) is 0.56707 and the p-value associated with this statistic is 7.371e-16 (extremely low).

For the third dataset “Free Disposability Hull” (fdh$eff), the Shapiro-Wilk test statistic (W) is 0.82434 and the p-value associated with this statistic is 1.144e-09 (very low).

In all cases, since the p-values are significantly lower than the usual significance level of 0.05, we reject the null hypothesis that the data follows a normal distribution. Therefore, we can conclude that none of the datasets passes the Shapiro-Wilk normality test and they do not follow a normal distribution.

Given the lack of normality in the data, it is essential to employ robust statistical techniques that allow for a reliable assessment of efficiency. In light of the results from the Shapiro-Wilk test indicating non-normality, Bootstrap emerges as a crucial tool. As a resampling technique that does not rely on strict assumptions about the distribution of data, Bootstrap offers an effective solution for estimating the distribution of key statistics such as efficiency and computing confidence intervals. Its ability to adapt to the data’s nature, even when it does not adhere to a normal distribution, provides a solid foundation for a rigorous and accurate analysis of efficiency in this context. ^20^^,^^21^

**Figure 1.**
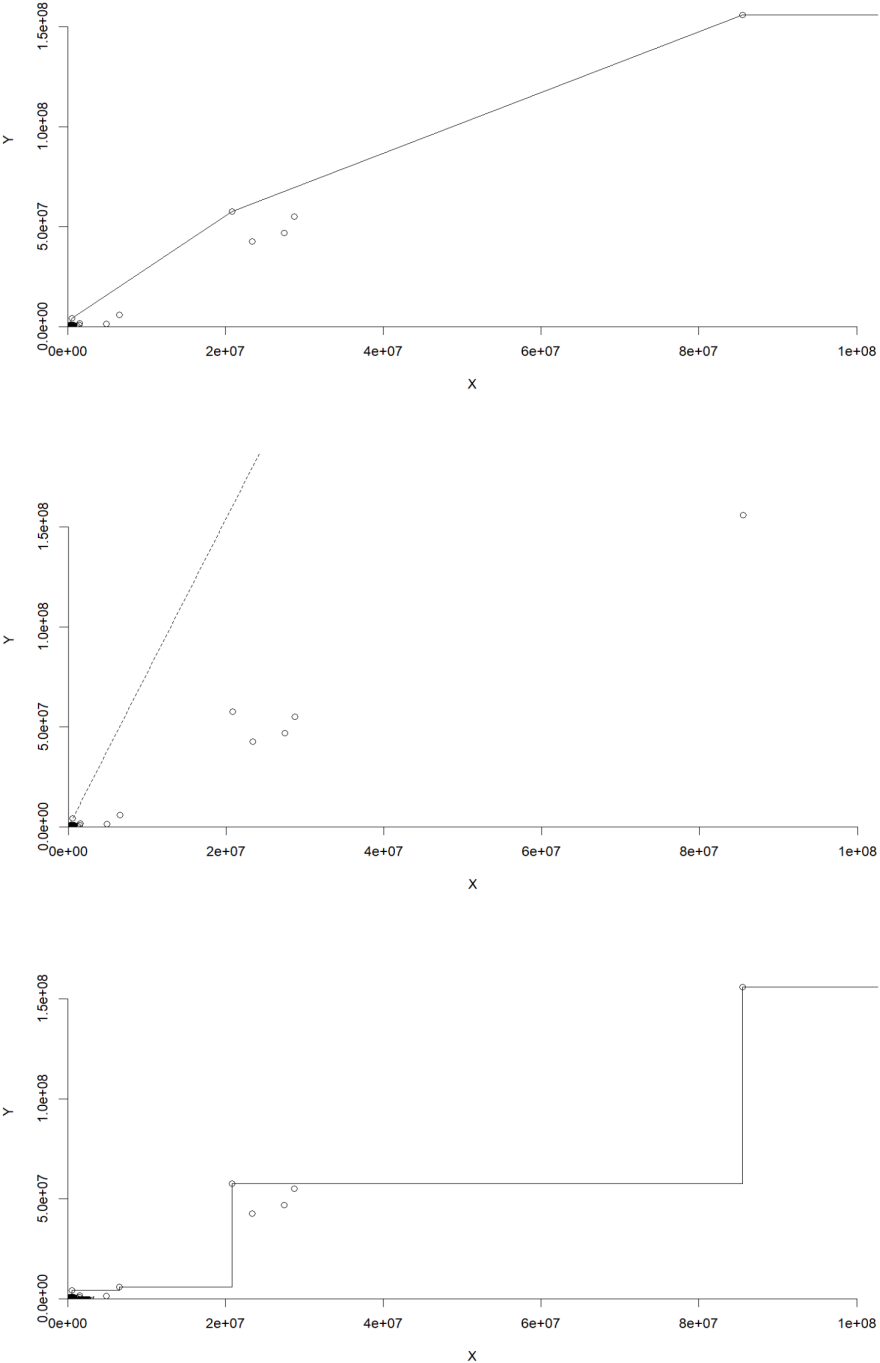
Efficiency distribution among farms under different technologies.

Table 10 and Figure 1, illustrate the distribution of efficiency levels across different technologies. Each cell represents the percentage of farms falling within a specific efficiency range for the respective technology. The table displays the distribution of efficiency levels across various ranges for three different technologies: VRS (Variable Returns to Scale), CRS (Constant Returns to Scale), and FDH (Free Disposal Hull). The table layout is similar to the one presented by Simar and Wilson. ^73^

**Table 10.** Summary of efficiencies. VRS, CRS technology and input orientated efficiency.

Eff range	VRS technology		CRS technology		FDH	
	Farm of #	%	Farm of #	%	Farm of #	%
0<= E <0.1			36	35.29		
0.1<= E <0.2			47	46.08		
0.2<= E <0.3	7	6.9	9	8.82		
0.3<= E <0.4	13	12.7	0	0.00	1	0.98
0.4<= E <0.5	31	30.4	2	1.96	6	5.88
0.5<= E <0.6	14	13.7	2	1.96	5	4.90
0.6<= E <0.7	7	6.9	0	0.00	11	10.78
0.7<= E <0.8	11	10.8	1	0.98	14	13.73
0.8<= E <0.9	8	7.8	1	0.98	15	14.71
0.9<= E <1	4	3.9	1	0.98	6	5.88
E ==1	7	6.9	3	2.94	44	43.14

**Table 11.** Bootstrap confidence intervals analysis for population parameters estimation.

VRS Technology				CRS Technology	
#	97.50%	2.50%		97.50%	2.50%
[1,]	0.734314	0.8588715		0.1492513	0.22571663
[2,]	0.2379865	0.2927612		0.04960946	0.07397499
[3,]	0.3974455	0.4801214		0.0917026	0.13777187
[4,]	0.3441167	0.3987667		0.03845313	0.05661101
[5,]	0.3708627	0.4538305		0.04911852	0.07361219
[6,]	0.5650645	0.7762285		0.12518336	0.1863432
[7,]	0.3592537	0.4060811		0.0538924	0.07931293
[8,]	0.4229691	0.5858517		0.07785097	0.11537871
[9,]	0.6202054	0.7221915		0.06699463	0.10069188
[10,]	0.4841339	0.5773089		0.04523652	0.06804455
[11,]	0.4283564	0.5474692		0.05448017	0.08321709
[12,]	0.4723178	0.5400138		0.05732273	0.08501577
[13,]	0.2614612	0.2968576		0.05868577	0.08915815
[14,]	0.1626129	0.2134181		0.06572995	0.09793264
[15,]	0.3445168	0.3899587		0.04471074	0.06713501
[16,]	0.4262115	0.5420283		0.07748749	0.11625805
[17,]	0.3405336	0.4220802		0.04040118	0.06049352
[18,]	0.2521135	0.3178658		0.06155695	0.09165241
[19,]	0.5492107	0.6742521		0.11867613	0.17689613
[20,]	0.3620185	0.4403513		0.04345827	0.06394605
[21,]	0.2663791	0.3175831		0.07098562	0.10673905
[22,]	0.4428699	0.4994617		0.06676287	0.09709695
[23,]	0.392298	0.4533156		0.07232646	0.10844461
[24,]	0.5282305	0.6733101		0.03262068	0.04902566
[25,]	0.1878778	0.2456769		0.06880526	0.10243414
[26,]	0.1778422	0.2072023		0.04867036	0.07275748
[27,]	0.4870985	0.5469948		0.10332508	0.15719843
[28,]	0.6661554	0.7872677		0.12735174	0.19051868
[29,]	0.7489755	0.9642545		0.12095816	0.18174418
[30,]	0.6924811	0.8048047		0.05488616	0.08219901
[31,]	0.3393679	0.4232449		0.13345056	0.19505656
[32,]	0.7445572	0.8811943		0.08377079	0.12745981
[33,]	0.4395966	0.5030473		0.1081356	0.1636377
[34,]	0.5770683	0.7281637		0.13316886	0.1994459
[35,]	0.3849385	0.4674695		0.08279154	0.12562813
[36,]	0.3846474	0.4594048		0.05874215	0.08744349
[37,]	0.5687409	0.6473213		0.05313533	0.07729841
[38,]	0.3001775	0.3707201		0.08730973	0.1273552
[39,]	0.2813466	0.318331		0.03512042	0.05071863
[40,]	0.6354041	0.8127566		0.07562446	0.11522808
[41,]	0.6494767	0.9719608		0.1579451	0.24169287
[42,]	0.7241378	0.8077864		0.10197259	0.15531257
[43,]	0.7133099	0.826717		0.117933	0.17698905
[44,]	0.474176	0.5338395		0.07292782	0.11001662
[45,]	0.3523753	0.4610211		0.09838257	0.15058469
[46,]	0.525413	0.611574		0.06413835	0.09787209
[47,]	0.3697758	0.4208191		0.0763362	0.11376024
[48,]	0.5613278	0.6443782		0.04937677	0.07457647
[49,]	0.5598437	0.772432		0.35498327	0.52989848
[50,]	0.5726299	0.7082447		0.06738397	0.10188178
[51,]	0.5425932	0.7019362		0.26056139	0.38683344
[52,]	0.4321841	0.5425175		0.16260801	0.24446609
[53,]	0.5697289	0.9676443		0.54659838	0.7938957
[54,]	0.5151711	0.7626995		0.45124464	0.69907337
[55,]	0.5955338	0.8891379		0.50081218	0.74958421
[56,]	0.6016284	0.9663721		0.57493752	0.81578109
[57,]	0.5370522	0.8188803		0.4708121	0.72443558
[58,]	0.3843784	0.4373357		0.09177044	0.13840801
[59,]	0.8208831	0.9305326		0.12606025	0.18995092
[60,]	0.3744484	0.4757413		0.13461959	0.19920591
[61,]	0.2998057	0.4260352		0.16960306	0.25922695
[62,]	0.35816	0.4050222		0.04745584	0.07156962
[63,]	0.2959887	0.4534394		0.27284986	0.40421992
[64,]	0.3590839	0.4116		0.0628196	0.08819324
[65,]	0.3430203	0.4606397		0.16543462	0.25238043
[66,]	0.3963086	0.4467608		0.06885001	0.10048332
[67,]	0.2912875	0.4093874		0.04273345	0.06361012
[68,]	0.2931068	0.3412685		0.02414241	0.03544619
[69,]	0.35741	0.4340573		0.09537608	0.1437752
[70,]	0.3561473	0.4289464		0.10513721	0.15510523
[71,]	0.3504989	0.4054093		0.05093963	0.07601636
[72,]	0.2324731	0.2867761		0.07886848	0.11816186
[73,]	0.4449375	0.6701119		0.33930217	0.51126479
[74,]	0.277722	0.3558817		0.10268267	0.15515434
[75,]	0.5686119	0.9659383		0.59260967	0.83120924
[76,]	0.3550062	0.437517		0.06799276	0.10115279
[77,]	0.3790457	0.4440742		0.07556346	0.11385764
[78,]	0.4833882	0.5491455		0.07664025	0.11278329
[79,]	0.427005	0.4779414		0.07531045	0.11423371
[80,]	0.3386361	0.4329602		0.16300382	0.23507594
[81,]	0.1657826	0.2238296		0.07674523	0.11583041
[82,]	0.2058686	0.2773171		0.10070824	0.1523901
[83,]	0.2365974	0.307009		0.1045076	0.15842976
[84,]	0.6037269	0.7962268		0.07249352	0.11077271
[85,]	0.2969126	0.3557266		0.08290021	0.12402695
[86,]	0.3888039	0.5133368		0.0480871	0.07311985
[87,]	0.5017564	0.7468721		0.0599779	0.09180817
[88,]	0.2875843	0.3799003		0.09000531	0.13381406
[89,]	0.3746959	0.4265403		0.07799345	0.11810869
[90,]	0.6597009	0.9669929		0.04809876	0.07161639
[91,]	0.3882102	0.4976015		0.10809376	0.16169388
[92,]	0.564242	0.6838645		0.02611752	0.03873205
[93,]	0.6999712	0.9619336		0.05412678	0.08277644
[94,]	0.8147992	0.946944		0.06050687	0.08951215
[95,]	0.354056	0.4161937		0.05052124	0.07568059
[96,]	0.5236482	0.7144467		0.07397693	0.11303726
[97,]	0.6079429	0.8053709		0.06147343	0.09388116
[98,]	0.4145848	0.4967461		0.03503671	0.05180126
[99,]	0.2835566	0.3288721		0.0281655	0.0424756
[100,]	0.4716659	0.5635434		0.05745141	0.08582234
[101,]	0.3447815	0.3913844		0.08287667	0.12485193
[102,]	0.3042556	0.3816386		0.03667248	0.0551207

VRS Technology: The majority of farms (35.29% to 46.08%) fall within the efficiency ranges of 0 to less than 0.2, indicating a relatively high level of efficiency. However, as the efficiency range increases beyond 0.5, the proportion of farms diminishes gradually, suggesting fewer farms operate at highly efficient levels under this technology.

CRS Technology: Similar to VRS, a significant proportion of farms (around 35% to 46%) exhibit high efficiency levels within the 0 to less than 0.2 range. Notably, there are instances where no farms achieve efficiency levels between 0.3 to less than 0.5, indicating potential inefficiencies for some farms under this technology.

FDH Technology: The distribution of farms across efficiency ranges under FDH displays a different pattern compared to VRS and CRS. While a notable proportion of farms operate at highly efficient levels (over 43%) when efficiency is exactly equal to 1, a substantial number of farms also demonstrate efficiency levels ranging from 0 to less than 0.2 (approximately 6.9% to 13.7%). Additionally, a sizable percentage of farms (over 10%) operate with efficiencies between 0.6 to less than 0.8, highlighting a varied efficiency landscape under this technology.

These intervals are constructed using bootstrap resampling, a technique for estimating the sampling distribution of a statistic by repeatedly resampling with replacement from the observed data. The resulting confidence intervals provide a range of plausible values for the population parameter estimated.

Upper Bound (97.5%), this value represents the upper limit of the confidence interval. It suggests that with 97.5% confidence, the true value of the parameter expected to be below this upper bound.

Lower Bound (2.5%), similarly, this value represents the lower limit of the confidence interval. With 97.5% confidence, the true value of the parameter expected to be above this lower bound.

The confidence levels (97.5% and 2.5%) indicate the probability that the true parameter lies within the calculated interval. In this case, a 95% confidence level commonly used, implying that there is a 95% probability that the true parameter falls within the calculated interval. The use of 97.5% and 2.5% might suggest a higher confidence level, which could be appropriate depending on the specific requirements of the analysis.

These confidence intervals are valuable in statistical inference, hypothesis testing, and parameter estimation. They provide a measure of uncertainty around the estimated parameter values, allowing researchers to make informed decisions and draw valid conclusions from their data.

In conclusion, the provided bootstrap confidence intervals offer valuable insights into the uncertainty associated with the estimated parameters, but their validity assessed through appropriate validation procedures.

## Conclusions

### Enhancing Efficiency and Policy Recommendations for Tlaxcala’s Dairy Farming Sector with Bootstrap Analysis

This study employed Data Envelopment Analysis (DEA) to scrutinize the efficiencies of Tlaxcala’s dairy farms, incorporating bootstrap analysis to validate and enhance the robustness of the findings. Utilizing the Variable Returns to Scale (VRS) model and DEAP version 2.1 software, the analysis ensured methodological transparency and adherence to DEA conventions.

### Key Insights from Data Analysis and Bootstrap

Preference for Input 1 (Cattle Investment): Bootstrap analysis reinforced the observation that many farms favored Input 1, the annual value of cattle investment, indicating its consistent cost-effectiveness across different samples.

Strong Option: Input 2 (Total Annual Cost): Bootstrap results confirmed the favorable status of Input 2, encompassing various costs like fuel and feeding, emphasizing its importance in maintaining efficiency across different scenarios.

Limited Popularity of Input 3 (Labor Costs): While not as prevalent, bootstrap analysis corroborated the observation that Input 3, representing labor costs, had limited influence on cost-effectiveness, suggesting consistent findings across multiple samples.

Farm-Specific Considerations: Bootstrap analysis provided robust evidence supporting the variability in optimal input choices among farms, reinforcing the importance of considering individual farm characteristics.

Insights from Radial and Slack Values: Bootstrap analysis enhanced the reliability of insights derived from radial and slack values, providing confidence in identifying areas for improvement and optimization.

### Policy Recommendations for Mexico’s Agricultural Sector

Support for Cattle Investment: Policies incentivizing and supporting cattle investment, backed by robust bootstrap analysis, can enhance efficiency and economic viability in dairy farming.

Comprehensive Cost Management: Bootstrap-supported policies focusing on comprehensive cost management, including fuel, feeding, and reproduction, can improve overall farm efficiency.

Optimization of Labor Costs: Bootstrap analysis reinforces the need for initiatives aimed at optimizing labor costs, such as training programs and technology adoption, to enhance labor efficiency on dairy farms.

Tailored Support: Policies informed by bootstrap analysis should be flexible and tailored to accommodate farm-specific factors, promoting efficiency based on robust evidence.

Promotion of Data-Driven Decision-Making: Bootstrap-supported policies promoting data-driven decision-making and technology adoption can optimize inputs and improve overall efficiency with greater confidence in the findings.

Encouragement of Optimization Strategies: Policies encouraging the adoption of practices aimed at reducing costs in identified areas, validated by bootstrap analysis, can lead to performance and sustainability improvements.

By integrating bootstrap analysis into policy recommendations, Mexico can advance towards a more efficient and sustainable agricultural landscape. Leveraging insights from both data analysis and robust bootstrap validation ensures that policies are evidence-based and capable of driving meaningful improvements in dairy farming efficiency and sustainability.

## Ethics statement

The protocol to carry out this research was reviewed and confirmed to proceed by the Colegio de Postgraduados (Institución de Enseñanza e Investigación en Ciencias Agrícolas). No formal ethical approval was required for this study as per the ‘Ley General de Protección de Datos Personales en Posesión de Sujeto Obligados’, regarding ethical approval requirements for this type of study. The questionnaire included a verbal statement requesting the consent of the producers in accordance with the provisions of the general law on the protection of personal data held by obligated subjects. Verbal as opposed to written consent was used because the aforementioned law does not require written consent to be bound by its compliance.

## Author contributions

Conceptualization: Carlos Zuniga

Methodology: Carlos Zuniga

Formal analysis: Carlos Zuniga, Jose Luis Jaramillo, Noel E. Blanco Roa

Investigation: Carlos Zuniga, Jose Luis Jaramillo, Noel E. Blanco Roa

Writing - original draft: Carlos Zuniga

Validation: Carlos Zuniga, Jose Luis Jaramillo, Noel E. Blanco Roa

Writing – review & editing: Carlos Zuniga, Jose Luis Jaramillo, Noel E. Blanco Roa

Data: Carlos Zuniga & Jose Luis Jaramillo

## Data availability

### Underlying data

Figshare: Data for: Inputs-Oriented VRS DEA in dairy farms, https://doi.org/10.6084/m9.figshare.21836133.v5. ^36^

This project contains the following underlying data:

• DataforDEAF1000R.cvs
• S1.csv (Suplementary Data for VRS Technology with Bootstrap DEA in R Studio)
• S2.csv (Suplementary 2 CRS Bootstrap DEA in R Studio)
• S3.csv (Dataset used for this study)

### Extended data

Figshare: Data for: Inputs-Oriented VRS DEA in dairy farms, https://doi.org/10.6084/m9.figshare.21836133.v5. ^36^

This project contains the following extended data:

• Questionnaire MilkProd.pdf (Questionnaire/interview guide translated to English)
• Questionnaire de campo_leche.pdf (Questionnaire/interview guide in Spanish)
• Table 1.csv
• Table 2.csv
• Table 3.scv
• Table 4.csv
• Table 5.csv
• Table 6.csv
• Table 7.csv
• Table 8.csv
• Table 9.csv
• Table 10.xlsx
• Table 11.xlsx
• Fig_1.tif
• Fig_2.tif
• Data for DEA F1000R.xlsx

Data are available under the terms of the Creative Commons Attribution 4.0 International license (CC-BY 4.0).
