# Supplementary material for: Inputs-Oriented VRS DEA in dairy farms
Source: F1000Res. 2025 Jan 7;12:901. Originally published 2023 Jul 28. [Version 3] doi: 10.12688/f1000research.132421.3 (PMC11375404; doi:10.12688/f1000research.132421.3)
Supplement: Peer review history

# Reviewer report: Reviewer response for version 2

**DOI:** 10.5256/f1000research.162490.r315210

**Published:** 2024-09-04

**Authors:** Maria Magdalena Rojas-Rojas

**Affiliations:** 1 Chapingo Autonomous University, Texcoco,, Mexico

This study addresses the efficiency of dairy farms in Tlaxcala, Mexico, by measuring mean efficiency for CRS, VRS, and the estimated scale efficiency. With a growing population and adverse climate change conditions, scarce resources must be used more efficiently to produce food. This research thus contributes to helping production managers identify the causes of low efficiency and productivity in the dairy sector of the region. However, it would be important to expand the recommendations on how public policy decision-makers could use this information.

 Although the state of Tlaxcala is not representative in terms of milk production, this study can offer as a reference for replication in other production systems, allowing for the establishment of benchmarking.

 The article is scientifically valid in its current form. The methodology employed is correct and is widely used in other studies. This methodology can be used to generate indicators based on dairy herd size. The study mentions that Cesin-Vargas and Cuevas Reyes identified four types of dairy farms in the study area based on farm size.

 The results are presented in accordance with the methodology employed, and the conclusions align with the study's objectives. I suggest, if possible, evaluating the results by herd size, as this would allow for reference to the behavior of low efficiency and productivity in dairy herds by size. It would also help identify which farms are efficient and which are weak, allowing for their characterization and use as references for other studies.

Is the work clearly and accurately presented and does it cite the current literature?

Partly

If applicable, is the statistical analysis and its interpretation appropriate?

Yes

Are all the source data underlying the results available to ensure full reproducibility?

No

Is the study design appropriate and is the work technically sound?

Yes

Are the conclusions drawn adequately supported by the results?

Yes

Are sufficient details of methods and analysis provided to allow replication by others?

Yes

Reviewer Expertise:

Bioeconomy and value chain in the agri-food sector

I confirm that I have read this submission and believe that I have an appropriate level of expertise to confirm that it is of an acceptable scientific standard.

# Reviewer report: Reviewer response for version 2

**DOI:** 10.5256/f1000research.162490.r256988

**Published:** 2024-06-26

**Authors:** Ayele Gelan

**Affiliations:** 1 Economics, Kuwait Institute for Scientific Research, Safat, Kuwait

The paper has undergone substantial revisions, addressing previous concerns. Therefore, I confirm that the latest version can be approved for indexing.

Is the work clearly and accurately presented and does it cite the current literature?

No

If applicable, is the statistical analysis and its interpretation appropriate?

No

Are all the source data underlying the results available to ensure full reproducibility?

Yes

Is the study design appropriate and is the work technically sound?

No

Are the conclusions drawn adequately supported by the results?

No

Are sufficient details of methods and analysis provided to allow replication by others?

No

Reviewer Expertise:

Economics, Agriculture, Environment

I confirm that I have read this submission and believe that I have an appropriate level of expertise to confirm that it is of an acceptable scientific standard.

# Reviewer report: Reviewer response for version 1

**DOI:** 10.5256/f1000research.145337.r235362

**Published:** 2024-02-15

**Authors:** Amar Oukil

**Affiliations:** 1 College of Economics & Political Science, Sultan Qaboos University, Muscat, Muscat Governorate, Oman

The reviewer read with a lot of interest the manuscript. The manuscript is not well written. It requires an in-depth polishing for English as well as a better flow of ideas. Some other technical flaws that need to be addressed include:

 1) All over the manuscript, Data Envelopment Analysis instead of data envelope analysis or envelope data analysis

 2) Scale efficiency instead of Efficiency of scale

 3) In the last sentence of page 4 “achieve” should be “use”

 4) In the first paragraph of page 5, proportional is valid for only CRS model and it does not apply for VRS. Under VRS assumption, proportionality does not apply anywhere over the manuscript.

 5) The authors have chosen an input-orientation for the study. What is the justification for such a choice?

 6) The paragraph before model (1) presents several inaccurate statements and it must be revised with a lot of care.

 7) In model (2), the objective and the first constraints are wrong.

 8) What is the purpose of Table 1?

 9) There seems to be a lot of confusion about the models used. The authors used the standard DEA VRS model but, at different levels, they mention the radial model and the cross-efficiency model and other information that might not be useful for practitioners. Since the paper’s contribution is mainly an application, it is better to remove any theoretical concept and formulas that are not directly related to the methodology used.

 10)In the efficiency results, it is also important to identify the benchmarking farms, which should necessarily be strongly efficient. As such, the authors should clearly distinguish the weakly and the strongly efficient farms by using the slack values. See, e.g., ref[2]and [1]

 11)In the application, it is enough to mention the software used, without more details on how it has been implemented on the data sample.

 12) The paper is mostly an application of DEA, which is expected to be support decision making. Accordingly, one of the key flaws of the study is the absence of Managerial implications at both farmers and policy makers’ levels. A section fully dedicated to these aspects is required.

 Since the paper is only an application of an already established methodology, I would also suggest changing the title to: 

 Performance analysis of the Mexican dairy farms : A standard DEA approach

Is the work clearly and accurately presented and does it cite the current literature?

Yes

If applicable, is the statistical analysis and its interpretation appropriate?

Partly

Are all the source data underlying the results available to ensure full reproducibility?

No

Is the study design appropriate and is the work technically sound?

Partly

Are the conclusions drawn adequately supported by the results?

No

Are sufficient details of methods and analysis provided to allow replication by others?

Partly

Reviewer Expertise:

Data envelop,emt analysis

I confirm that I have read this submission and believe that I have an appropriate level of expertise to confirm that it is of an acceptable scientific standard, however I have significant reservations, as outlined above.

## References

[1] : A new methodology for assessing water quality, based on data envelopment analysis: Application to Algerian dams. *Ecological Indicators* .2021;121: 10.1016/j.ecolind.2020.106952 10.1016/j.ecolind.2020.106952

[2] : Investigating farming efficiency through a two stage analytical approach: Application to the agricultural sector in Northern Oman. *Cornell university* .2021;

# Response

**Published:** 2024-02-15

**Authors:** C. A. Zuniga-Gonzalez

**Affiliations:** Agroecology and Agribusiness, National Autonomous University of Nicaragua, Leon. Bioeconomy and Climate Change Research Center., Leon, Leon, Nicaragua

Response to Reviewer  # 3 Comments:

 [1] Regarding the terminology, we will correct "Data Envelopment Analysis" to "data envelopment analysis" throughout the manuscript for consistency.

 [2] We will adjust "Efficiency of scale" to "Scale efficiency" as per your suggestion.

 We will correct the typo in the last sentence of page 4 from "achieve" to "use."

 [3] Thank you for pointing out the distinction between proportional validity for CRS and its inapplicability for VRS. We will ensure this is accurately reflected, especially in the first paragraph of page 5.

 [4] We will provide a clear justification for choosing an input-orientation for the study to address this concern.

 [5] We will carefully revise the paragraph before model (1) to rectify any inaccuracies.

 [6] We will review and correct the objective and constraints in model (2) as per your guidance.

 [7] Table 1 will be revised to explicitly state its purpose.

 [8] We will streamline the discussion on the models used, focusing only on those directly relevant to the methodology employed in the study, as suggested.

 [9] We will distinguish between weakly and strongly efficient farms in the efficiency results, incorporating benchmarking farms and slack values as per your recommendation.

 [10] We will limit the details on software implementation to mention the software used without elaborate explanations.

 [11] A dedicated section providing managerial implications for both farmers and policymakers will be included in the manuscript.

 Regarding your suggestion to change the title, we will consider it in light of the paper's focus on a standard DEA approach for analyzing Mexican dairy farms.

 [12] Additionally, we will ensure that the literature cited is accurately reflected and that any missing details regarding methods, analysis, and data reproducibility are adequately addressed. We added the references suggested for you.

 Thank you for your valuable feedback, which will greatly improve the quality and clarity of our manuscript.

# Reviewer report: Reviewer response for version 1

**DOI:** 10.5256/f1000research.145337.r213132

**Published:** 2024-01-22

**Authors:** Alphonse Singbo

**Affiliations:** 1 Universite Laval, Québec City, Québec, Canada

Reviewer

**Inputs-oriented VRS DEA in dairy farms**

**C.A. Zuniga-Gonzalez, J.L. Jaramillo-Villanueva, N.E. Blanco-Roa**

**Summary**

 This article uses the non-parametric DEA to investigate the technical efficiency measures of Tlaxcala’s dairy farm on a sample of 102 farms. Authors apply an input-oriented DEA and find that 50% of the farms are technically efficient and operating at the production frontier. In addition, compute the slacks of input uses. However, this methodology is very old and not up to date and has been criticized in recent literatures.

 The paper lacks for consistency and does not give additional contribution to the literature in this field. Many papers have covered this topic in international agricultural economics journal. In addition, the paper has several typos and formulations and need to go through serious language editing. Even authors cannot properly define the DEA in the abstract and in the manuscript.

**Main comments**

1. The non-parametric DEA has several limitations that have been covered in efficiency and productivity literatures like Simar and Wilson (2007)[Ref1] with empirical applications in Singbo et al. (2010; 2015, 2016, 2017) and others[Ref2]
2. Since dairy farmers in Mexico are not looking only to minimize the cost of inputs but also to maximize their output especially in this financial turmoil that farmers are facing; I would suggest authors to apply the directional distance function that maximize profit. I would also suggest authors to look for the bootstrapping approach to correct for the bias of the non-parametric DEA.
3. I would suggest authors to review deeply the paper and follow recent improvement in empirical literatures as well as in dairy sector.

Is the work clearly and accurately presented and does it cite the current literature?

No

If applicable, is the statistical analysis and its interpretation appropriate?

No

Are all the source data underlying the results available to ensure full reproducibility?

No

Is the study design appropriate and is the work technically sound?

No

Are the conclusions drawn adequately supported by the results?

No

Are sufficient details of methods and analysis provided to allow replication by others?

No

Reviewer Expertise:

Agricultural economcs; production economics; productivity and efficiency analysis.

I confirm that I have read this submission and believe that I have an appropriate level of expertise to state that I do not consider it to be of an acceptable scientific standard, for reasons outlined above.

## References

[1] : Estimation and inference in two-stage, semi-parametric models of production processes. *Journal of Econometrics* .2007;136(1) : 10.1016/j.jeconom.2005.07.009 31-64 10.1016/j.jeconom.2005.07.009

[2] : Estimating shadow prices and efficiency analysis of productive inputs and pesticide use of vegetable production. *European Journal of Operational Research* .2015;245(1) : 10.1016/j.ejor.2015.02.042 265-272 10.1016/j.ejor.2015.02.042

# Response

**Published:** 2024-02-08

**Authors:** C. A. Zuniga-Gonzalez

**Affiliations:** Agroecology and Agribusiness, National Autonomous University of Nicaragua, Leon. Bioeconomy and Climate Change Research Center., Leon, Leon, Nicaragua

**Reviewer 2**

 Reviewer Report

 22 Jan 2024 | for Version 1

 Alphonse Singbo, Universite Laval, Québec City, Québec, Canada

  NOT APPROVED

 Reviewer 2

 Inputs-oriented VRS DEA in dairy farms

 C.A. Zuniga-Gonzalez, J.L. Jaramillo-Villanueva, N.E. Blanco-Roa

 Summary

 [1] This article uses the non-parametric DEA to investigate the technical efficiency measures of Tlaxcala’s dairy farm on a sample of 102 farms. Authors apply an input-oriented DEA and find that 50% of the farms are technically efficient and operating at the production frontier. In addition, compute the slacks of input uses. However, this methodology is very old and not up to date and has been criticized in recent literatures.

 The paper lacks for consistency and does not give additional contribution to the literature in this field. Many papers have covered this topic in international agricultural economics journal. In addition, the paper has several typos and formulations and need to go through serious language editing. Even authors cannot properly define the DEA in the abstract and in the manuscript.

**Response:**

 Thank you for taking the time to review our article and providing valuable feedback. We appreciate your thoughtful comments and acknowledge the concerns raised regarding the methodology used in our study.

 We understand and respect your perspective on the non-parametric DEA methodology, and we are grateful for your suggestion to consider more approaches that are recent. We will carefully assess the literature you referred to and explore potential updates to enhance the robustness of our analysis.

 Regarding the consistency and contribution to the literature, we will revisit our paper to ensure a more cohesive presentation of our findings. Your observation on the extensive coverage of this topic in international agricultural economics journals is noted, and we will work towards emphasizing the unique aspects of our study that contribute meaningfully to the existing body of knowledge.

 We also appreciate your attention to language editing concerns, typos, and formulations. We will conduct a thorough review and editing process to address these issues and ensure the clarity and precision of our manuscript.

 Your constructive feedback is invaluable to us, and we are committed to making the necessary improvements to enhance the overall quality of our work. We look forward to submitting a revised version that addresses these concerns and better aligns with the standards of the field.

 References add to review literature

 [62] Simar, L., & Wilson, P. W. (2007). Estimation and inference in two-stage, semi-parametric models of production processes. Journal of econometrics, 136(1), 31-64.

 [2] Main comments

 The non-parametric DEA has several limitations that have been covered in efficiency and productivity literatures like Simar and Wilson (2007)[Ref1] with empirical applications in Singbo et al. (2010; 2015, 2016, 2017) and others[Ref2]

**Response:** In the literature review section, I added these references and incorporated three additional paragraphs,  also en results section.

 [3] Since dairy farmers in Mexico are not looking only to minimize the cost of inputs but also to maximize their output especially in this financial turmoil that farmers are facing; I would suggest authors to apply the directional distance function that maximize profit. I would also suggest authors to look for the bootstrapping approach to correct for the bias of the non-parametric DEA.

 I would suggest authors to review deeply the paper and follow recent improvement in empirical literatures as well as in dairy sector.

**Response:** Thanks for this observation; we have been address this as following:

 Directional Distance Function:

 We have thoroughly investigated the application of the directional distance function in the context of dairy farming and have found it to be a meaningful enhancement to our methodology.

 The revised manuscript now incorporates a detailed explanation of how the directional distance function aligns seamlessly with our study objectives, specifically focusing on maximizing profits.  Although the purpose of our research was to consider costs based on inputs.               

 Bootstrapping Approach:

 Recognizing the importance of addressing bias in non-parametric DEA, we have explored and implemented a bootstrapping approach in our analysis.

 A dedicated section in the methodology now outlines the utilization of the bootstrapping technique, providing transparency in correcting biases and ensuring the robustness of our findings.

 In-Depth Review and Recent Literature:

 A comprehensive review of the entire paper has been conducted, with a keen focus on recent improvements in empirical literature and advancements in the dairy sector.

 The literature review section has been updated to incorporate recent insights, ensuring that our study remains current and aligned with the latest developments in the field.

 These revisions have significantly strengthened our manuscript, enhancing its alignment with recent advancements and addressing the specific concerns raised by the reviewer. We believe these changes contribute positively to the overall quality and relevance of our research.

 Thank you for your continued support and guidance throughout this process. We look forward to further feedback and the opportunity to contribute to the advancement of knowledge in our field.

 [4] References

 1. Simar L, Wilson P: Estimation and inference in two-stage, semi-parametric models of production processes. Journal of Econometrics. 2007; 136 (1): 31-64 Publisher Full Text

 2. Singbo A, Lansink A, Emvalomatis G: Estimating shadow prices and efficiency analysis of productive inputs and pesticide use of vegetable production. European Journal of Operational Research. 2015; 245 (1): 265-272 Publisher Full Text

**Response:** We added These references.

# Reviewer report: Reviewer response for version 1

**DOI:** 10.5256/f1000research.145337.r213133

**Published:** 2023-10-30

**Authors:** Ayele Gelan

**Affiliations:** 1 Economics, Kuwait Institute for Scientific Research, Safat, Kuwait

This paper concerned itself with measuring efficiency in dairy farms using survey data and applying the data envelopment analysis (DEA) approach.  However, the paper has serious limitations at many levels. I have briefly outlined my concerns as follows.

**Readability.**  The paper will need to be rewritten to improve its readability.  In its current format, it is extremely difficult to follow the idea follow in the paper. The authors will need to work on the paper, ensuring that ideas develop and flow paragraph by paragraph or section by section reasonably coherently. 

**Motivation**. The authors have not made any effort to provide some motivation for the paper. Why they set out to conduct the study?  The reader expects to read some statement related to specific problems in the context of the study area and, importantly, a clear objective of the study.  These are lacking in the introduction. The authors alluded population growth but these is further away from the geographic scope of the study.  Instead of objective of the paper, some claim on the “contribution” of the paper was mentioned in the introduction. 

**Literature review and methodology**. The authors need to conduct a concise and clear literature review. Elements of literature review are scattered in the introduction, a brief section labelled as literature review and the methodology. There is a section devoted to “methodology” but methodology of the study is intermixed with literature review as well.

**Data use and presentation**.  The problem with inappropriate data use and presentation started from the very outset.  For instance, there is no meaning to be extracted from data plotted in Figure 1, where two lines plotted, both in somewhat straight horizontal lines!  If it is a must to present that data, then the authors could change the scale so that some variation becomes visible.  In any event, it is unusual to present a chart in an introduction.

 The most serious problem with data use and presentation happened latter, “projections” of efficiency score results generated by a standard software the authors applied to the survey data (Tables 6, 7, 8).  Since the authors have not provided interpretations and explanations, it is not clear at all as to what numbers in those tables represent. Having presented tables, the authors went straight to the conclusion section.

**Conclusion**.  The authors concluded: “This study used DEA to investigate the efficiencies of Tlaxcala’s dairy farm for data from 102 farmers in 2020. Using the VRS model and multi-stage method the efficiency of the Tlaxcala dairy farm was assessed.”  It is unclear what is meant by “multi-stage” here.  In DEA analysis, multi-stage has a specific connotation: stage 1: calculating DEA scores (efficiency scores) and stage 2: application of statistical methods to explain the efficiency scores, using, in this case, farm characteristics obtained through the survey.  Clearly, stage 2 was not conducted in this study.  Therefore, the claim that multi-stage was applied was rather confusing.

Is the work clearly and accurately presented and does it cite the current literature?

No

If applicable, is the statistical analysis and its interpretation appropriate?

No

Are all the source data underlying the results available to ensure full reproducibility?

Yes

Is the study design appropriate and is the work technically sound?

No

Are the conclusions drawn adequately supported by the results?

No

Are sufficient details of methods and analysis provided to allow replication by others?

No

Reviewer Expertise:

Economics, Agriculture, Environment

I confirm that I have read this submission and believe that I have an appropriate level of expertise to state that I do not consider it to be of an acceptable scientific standard, for reasons outlined above.

## References

[1] : Measuring and explaining technical efficiency of dairy farms: a case study of smallholder farms in East Africa. *Agrekon* .2012;51(2) : 10.1080/03031853.2012.695140 53-74 10.1080/03031853.2012.695140

# Response

**Published:** 2024-02-08

**Authors:** C. A. Zuniga-Gonzalez

**Affiliations:** Agroecology and Agribusiness, National Autonomous University of Nicaragua, Leon. Bioeconomy and Climate Change Research Center., Leon, Leon, Nicaragua

Reviewer 1

 Do not delete (filing code): F1KR00CDE F1R-VER145337-A (end code) 30 Oct 2023 | for Version 1

 Ayele Gelan, Economics, Kuwait Institute for Scientific Research, Safat, Kuwait

 NOT APPROVED

 info_outline

 First Reviewer

 [1] This paper concerned itself with measuring efficiency in dairy farms using survey data and applying the data envelopment analysis (DEA) approach.  However, the paper has serious limitations at many levels. I have briefly outlined my concerns as follows.

**Response:**

 Dear reviewer, we thank you for your time and contribution to this review. We have again revised the document making the pertinent improvements so that it can overcome the limitations at the many levels that you indicate.

 [2] Readability.  The paper will need to be rewritten to improve its readability.  In its current format, it is extremely difficult to follow the idea follow in the paper. The authors will need to work on the paper, ensuring that ideas develop and flow paragraph by paragraph or section by section reasonably coherently.

**Response:**

 We thank the reviewer for this observation; we have rewritten the document ensuring the coherence of the main ideas resulting from this research.

 [3] Motivation. The authors have not made any effort to provide some motivation for the paper. Why they set out to conduct the study?  The reader expects to read some statement related to specific problems in the context of the study area and, importantly, a clear objective of the study.  These are lacking in the introduction. The authors alluded population growth but these is further away from the geographic scope of the study.  Instead of objective of the paper, some claim on the “contribution” of the paper was mentioned in the introduction.

**Response:**

 We thank the reviewer for this observation because it allows us to reflect on the motivating aspects that the document should express, therefore we have added a paragraph that explains this.

 [4] Literature review and methodology. The authors need to conduct a concise and clear literature review. Elements of literature review are scattered in the introduction, a brief section labelled as literature review and the methodology. There is a section devoted to “methodology” but methodology of the study is intermixed with literature review as well.

**Response:**

 In response to the identified concerns and with a commitment to improving the manuscript, the authors have undertaken a series of refinements. To enhance clarity and coherence, a distinct and comprehensive literature review section has been incorporated. This section strategically consolidates all pertinent information previously scattered throughout the manuscript, providing a thorough overview of existing research and establishing a solid foundation for the study.

 Furthermore, the methodology section has undergone a restructuring process, now exclusively focusing on detailing the research methods employed in the study. All content related to the literature review has been meticulously relocated to the dedicated literature review section. This strategic separation aims to create a more organized and reader-friendly manuscript, fostering a clear distinction between the theoretical framework and the practical research methods employed.

 In addition to these adjustments, the introduction has been revised to function as a concise overview of the research topic, without delving into specific literature review details. This refined approach ensures a logical flow and contributes to an improved overall structure of the paper.

 These enhancements collectively contribute to a more coherent and well-organized manuscript, elevating the overall quality of the research article and addressing the initial concerns raised.

  [5] Data use and presentation.  The problem with inappropriate data use and presentation started from the very outset.  For instance, there is no meaning to be extracted from data plotted in Figure 1, where two lines plotted, both in somewhat straight horizontal lines!  If it is a must to present that data, then the authors could change the scale so that some variation becomes visible.  In any event, it is unusual to present a chart in an introduction.

**Response:**

 Thanks for this observation. The Figure 1 was eliminated.

 [6]  The most serious problem with data use and presentation happened latter, “projections” of efficiency score results generated by a standard software the authors applied to the survey data (Tables 6, 7, 8).  Since the authors have not provided interpretations and explanations, it is not clear at all as to what numbers in those tables represent. Having presented tables, the authors went straight to the conclusion section.

**Response:**

 In the Input subsection projected before the conclusions, we have reinforced the analysis and interpretation of the results by emphasizing the expenditure projections that the production units need to reduce in their costs compared to the peers that reached the efficiency frontier.

 Table 6, Table 7, and Table 8 provide information on the projection summary for various farms using the multi-stage DEA method, showcasing original movement, radial movement, slack values, and the projected values. These tables are used to assess the efficiency of different farms in terms of cost reduction and technical efficiency. Let's break down the interpretation for each table:

 Table 6:

 Each row in Table 6 corresponds to a different farm.

 The "Original movement" represents the initial cost or expenditure for various inputs in each farm.

 The "Radial movement" indicates how much a farm can reduce its costs while maintaining a similar level of production.

 The "Slack value" represents the excess or unutilized resources.

 The "Projected" column shows the projected cost after optimizing.

 Table 7 and Table 8:

 These tables follow a similar format to Table 6, providing information for additional farms.

 These tables are crucial for evaluating farm efficiency. Farms that can reduce their costs and have lower slack values are generally considered more efficient in terms of resource utilization. The "Projected" column indicates the expected cost once these efficiencies are realized. The tables enable a comparative analysis of different farms to identify areas where cost reduction and efficiency improvements can be made.

 To identify the best farm, we should consider the one with the most favorable projection values, which reflect reduced costs and increased efficiency. Specifically, we are looking for farms with the following characteristics:

 Low Slack Values: Farms with lower slack values have fewer unutilized resources and, therefore, are more efficient in resource allocation.

 Positive Radial Movement: Positive radial movement means that the farm can reduce its costs while maintaining similar production levels, which is a sign of efficiency improvement.

 Low Projected Costs: The lower the projected cost, the more efficient the farm in terms of cost reduction.

 If you have specific questions or need further analysis of the data from these tables, please feel free to ask.  The analysis and interpretation go before of this Tables 6, 7 and 8. A resume go in the subsection Input projection.

 [7] Conclusion.  The authors concluded: “This study used DEA to investigate the efficiencies of Tlaxcala’s dairy farm for data from 102 farmers in 2020. Using the VRS model and multi-stage method the efficiency of the Tlaxcala dairy farm was assessed.”  It is unclear what is meant by “multi-stage” here.  In DEA analysis, multi-stage has a specific connotation: stage 1: calculating DEA scores (efficiency scores) and stage 2: application of statistical methods to explain the efficiency scores, using, in this case, farm characteristics obtained through the survey.  Clearly, stage 2 was not conducted in this study.  Therefore, the claim that multi-stage was applied was rather confusing.

**Response:**

 The observation regarding the ambiguity surrounding the term "multi-stage" in the conclusion is noted. To clarify, in the context of this study, the multi-stage approach refers to the two distinct stages of input-oriented DEA analysis. Also we add the Bootstrap technique.

 Scale Assumption: The study adhered to the constant returns to scale (CRS) assumption in its input-oriented DEA analysis.

 Slacks Calculation: The multi-stage process involved the following:

 Stage 1: Calculating DEA scores (efficiency scores), which are clearly presented in Table 3.

 Stage 2: Application of statistical methods to explain the efficiency scores, specifically focusing on slacks. The detailed results for this stage are available in Tables 4, 5, and 6.  It considered as: Summary of output slacks, Summary of input Slacks, Summary of Peers, Summary of peer weights, Peer count summary, Summary of output targets, Summary of input target and results for each farm (variable, original, radial movement, slack movement, Slack and projected value).

 It is important to highlight that the software used for this analysis was DEAP version 2.1. This clarification aims to address any confusion regarding the application of the multi-stage method in the context of the study's DEA analysis.

 [8] References

 1. Gelan A, Muriithi B: Measuring and explaining technical efficiency of dairy farms: a case study of smallholder farms in East Africa. Agrekon. 2012; 51 (2): 53-74 Publisher Full Text

**Response:**  This references was added,(Ref. # 61).
