# Supplementary material for: Inputs-Oriented VRS DEA in dairy farms
Source: F1000Res. 2024 Mar 18;12:901. Originally published 2023 Jul 28. [Version 2] doi: 10.12688/f1000research.132421.2 (PMC11375404; doi:10.12688/f1000research.132421.2)
Supplement: Peer review history

# Response

**Published:** 2024-02-15

**Authors:** C. A. Zuniga-Gonzalez

**Affiliations:** Agroecology. ECAV. CIByCC., National Autonomous University of Nicaragua, Leon. Bioeconomy and Climate Change Research Center., Leon, Leon, Nicaragua

Response to Reviewer  # 3 Comments:

 [1] Regarding the terminology, we will correct "Data Envelopment Analysis" to "data envelopment analysis" throughout the manuscript for consistency.

 [2] We will adjust "Efficiency of scale" to "Scale efficiency" as per your suggestion.

 We will correct the typo in the last sentence of page 4 from "achieve" to "use."

 [3] Thank you for pointing out the distinction between proportional validity for CRS and its inapplicability for VRS. We will ensure this is accurately reflected, especially in the first paragraph of page 5.

 [4] We will provide a clear justification for choosing an input-orientation for the study to address this concern.

 [5] We will carefully revise the paragraph before model (1) to rectify any inaccuracies.

 [6] We will review and correct the objective and constraints in model (2) as per your guidance.

 [7] Table 1 will be revised to explicitly state its purpose.

 [8] We will streamline the discussion on the models used, focusing only on those directly relevant to the methodology employed in the study, as suggested.

 [9] We will distinguish between weakly and strongly efficient farms in the efficiency results, incorporating benchmarking farms and slack values as per your recommendation.

 [10] We will limit the details on software implementation to mention the software used without elaborate explanations.

 [11] A dedicated section providing managerial implications for both farmers and policymakers will be included in the manuscript.

 Regarding your suggestion to change the title, we will consider it in light of the paper's focus on a standard DEA approach for analyzing Mexican dairy farms.

 [12] Additionally, we will ensure that the literature cited is accurately reflected and that any missing details regarding methods, analysis, and data reproducibility are adequately addressed. We added the references suggested for you.

 Thank you for your valuable feedback, which will greatly improve the quality and clarity of our manuscript.

# Response

**Published:** 2024-02-08

**Authors:** C. A. Zuniga-Gonzalez

**Affiliations:** Agroecology. ECAV. CIByCC., National Autonomous University of Nicaragua, Leon. Bioeconomy and Climate Change Research Center., Leon, Leon, Nicaragua

**Reviewer 2**

 Reviewer Report

 22 Jan 2024 | for Version 1

 Alphonse Singbo, Universite Laval, Québec City, Québec, Canada

  NOT APPROVED

 Reviewer 2

 Inputs-oriented VRS DEA in dairy farms

 C.A. Zuniga-Gonzalez, J.L. Jaramillo-Villanueva, N.E. Blanco-Roa

 Summary

 [1] This article uses the non-parametric DEA to investigate the technical efficiency measures of Tlaxcala’s dairy farm on a sample of 102 farms. Authors apply an input-oriented DEA and find that 50% of the farms are technically efficient and operating at the production frontier. In addition, compute the slacks of input uses. However, this methodology is very old and not up to date and has been criticized in recent literatures.

 The paper lacks for consistency and does not give additional contribution to the literature in this field. Many papers have covered this topic in international agricultural economics journal. In addition, the paper has several typos and formulations and need to go through serious language editing. Even authors cannot properly define the DEA in the abstract and in the manuscript.

**Response:**

 Thank you for taking the time to review our article and providing valuable feedback. We appreciate your thoughtful comments and acknowledge the concerns raised regarding the methodology used in our study.

 We understand and respect your perspective on the non-parametric DEA methodology, and we are grateful for your suggestion to consider more approaches that are recent. We will carefully assess the literature you referred to and explore potential updates to enhance the robustness of our analysis.

 Regarding the consistency and contribution to the literature, we will revisit our paper to ensure a more cohesive presentation of our findings. Your observation on the extensive coverage of this topic in international agricultural economics journals is noted, and we will work towards emphasizing the unique aspects of our study that contribute meaningfully to the existing body of knowledge.

 We also appreciate your attention to language editing concerns, typos, and formulations. We will conduct a thorough review and editing process to address these issues and ensure the clarity and precision of our manuscript.

 Your constructive feedback is invaluable to us, and we are committed to making the necessary improvements to enhance the overall quality of our work. We look forward to submitting a revised version that addresses these concerns and better aligns with the standards of the field.

 References add to review literature

 [62] Simar, L., & Wilson, P. W. (2007). Estimation and inference in two-stage, semi-parametric models of production processes. Journal of econometrics, 136(1), 31-64.

 [2] Main comments

 The non-parametric DEA has several limitations that have been covered in efficiency and productivity literatures like Simar and Wilson (2007)[Ref1] with empirical applications in Singbo et al. (2010; 2015, 2016, 2017) and others[Ref2]

**Response:** In the literature review section, I added these references and incorporated three additional paragraphs,  also en results section.

 [3] Since dairy farmers in Mexico are not looking only to minimize the cost of inputs but also to maximize their output especially in this financial turmoil that farmers are facing; I would suggest authors to apply the directional distance function that maximize profit. I would also suggest authors to look for the bootstrapping approach to correct for the bias of the non-parametric DEA.

 I would suggest authors to review deeply the paper and follow recent improvement in empirical literatures as well as in dairy sector.

**Response:** Thanks for this observation; we have been address this as following:

 Directional Distance Function:

 We have thoroughly investigated the application of the directional distance function in the context of dairy farming and have found it to be a meaningful enhancement to our methodology.

 The revised manuscript now incorporates a detailed explanation of how the directional distance function aligns seamlessly with our study objectives, specifically focusing on maximizing profits.  Although the purpose of our research was to consider costs based on inputs.               

 Bootstrapping Approach:

 Recognizing the importance of addressing bias in non-parametric DEA, we have explored and implemented a bootstrapping approach in our analysis.

 A dedicated section in the methodology now outlines the utilization of the bootstrapping technique, providing transparency in correcting biases and ensuring the robustness of our findings.

 In-Depth Review and Recent Literature:

 A comprehensive review of the entire paper has been conducted, with a keen focus on recent improvements in empirical literature and advancements in the dairy sector.

 The literature review section has been updated to incorporate recent insights, ensuring that our study remains current and aligned with the latest developments in the field.

 These revisions have significantly strengthened our manuscript, enhancing its alignment with recent advancements and addressing the specific concerns raised by the reviewer. We believe these changes contribute positively to the overall quality and relevance of our research.

 Thank you for your continued support and guidance throughout this process. We look forward to further feedback and the opportunity to contribute to the advancement of knowledge in our field.

 [4] References

 1. Simar L, Wilson P: Estimation and inference in two-stage, semi-parametric models of production processes. Journal of Econometrics. 2007; 136 (1): 31-64 Publisher Full Text

 2. Singbo A, Lansink A, Emvalomatis G: Estimating shadow prices and efficiency analysis of productive inputs and pesticide use of vegetable production. European Journal of Operational Research. 2015; 245 (1): 265-272 Publisher Full Text

**Response:** We added These references.

# Response

**Published:** 2024-02-08

**Authors:** C. A. Zuniga-Gonzalez

**Affiliations:** Agroecology. ECAV. CIByCC., National Autonomous University of Nicaragua, Leon. Bioeconomy and Climate Change Research Center., Leon, Leon, Nicaragua

Reviewer 1

 Do not delete (filing code): F1KR00CDE F1R-VER145337-A (end code) 30 Oct 2023 | for Version 1

 Ayele Gelan, Economics, Kuwait Institute for Scientific Research, Safat, Kuwait

 NOT APPROVED

 info_outline

 First Reviewer

 [1] This paper concerned itself with measuring efficiency in dairy farms using survey data and applying the data envelopment analysis (DEA) approach.  However, the paper has serious limitations at many levels. I have briefly outlined my concerns as follows.

**Response:**

 Dear reviewer, we thank you for your time and contribution to this review. We have again revised the document making the pertinent improvements so that it can overcome the limitations at the many levels that you indicate.

 [2] Readability.  The paper will need to be rewritten to improve its readability.  In its current format, it is extremely difficult to follow the idea follow in the paper. The authors will need to work on the paper, ensuring that ideas develop and flow paragraph by paragraph or section by section reasonably coherently.

**Response:**

 We thank the reviewer for this observation; we have rewritten the document ensuring the coherence of the main ideas resulting from this research.

 [3] Motivation. The authors have not made any effort to provide some motivation for the paper. Why they set out to conduct the study?  The reader expects to read some statement related to specific problems in the context of the study area and, importantly, a clear objective of the study.  These are lacking in the introduction. The authors alluded population growth but these is further away from the geographic scope of the study.  Instead of objective of the paper, some claim on the “contribution” of the paper was mentioned in the introduction.

**Response:**

 We thank the reviewer for this observation because it allows us to reflect on the motivating aspects that the document should express, therefore we have added a paragraph that explains this.

 [4] Literature review and methodology. The authors need to conduct a concise and clear literature review. Elements of literature review are scattered in the introduction, a brief section labelled as literature review and the methodology. There is a section devoted to “methodology” but methodology of the study is intermixed with literature review as well.

**Response:**

 In response to the identified concerns and with a commitment to improving the manuscript, the authors have undertaken a series of refinements. To enhance clarity and coherence, a distinct and comprehensive literature review section has been incorporated. This section strategically consolidates all pertinent information previously scattered throughout the manuscript, providing a thorough overview of existing research and establishing a solid foundation for the study.

 Furthermore, the methodology section has undergone a restructuring process, now exclusively focusing on detailing the research methods employed in the study. All content related to the literature review has been meticulously relocated to the dedicated literature review section. This strategic separation aims to create a more organized and reader-friendly manuscript, fostering a clear distinction between the theoretical framework and the practical research methods employed.

 In addition to these adjustments, the introduction has been revised to function as a concise overview of the research topic, without delving into specific literature review details. This refined approach ensures a logical flow and contributes to an improved overall structure of the paper.

 These enhancements collectively contribute to a more coherent and well-organized manuscript, elevating the overall quality of the research article and addressing the initial concerns raised.

  [5] Data use and presentation.  The problem with inappropriate data use and presentation started from the very outset.  For instance, there is no meaning to be extracted from data plotted in Figure 1, where two lines plotted, both in somewhat straight horizontal lines!  If it is a must to present that data, then the authors could change the scale so that some variation becomes visible.  In any event, it is unusual to present a chart in an introduction.

**Response:**

 Thanks for this observation. The Figure 1 was eliminated.

 [6]  The most serious problem with data use and presentation happened latter, “projections” of efficiency score results generated by a standard software the authors applied to the survey data (Tables 6, 7, 8).  Since the authors have not provided interpretations and explanations, it is not clear at all as to what numbers in those tables represent. Having presented tables, the authors went straight to the conclusion section.

**Response:**

 In the Input subsection projected before the conclusions, we have reinforced the analysis and interpretation of the results by emphasizing the expenditure projections that the production units need to reduce in their costs compared to the peers that reached the efficiency frontier.

 Table 6, Table 7, and Table 8 provide information on the projection summary for various farms using the multi-stage DEA method, showcasing original movement, radial movement, slack values, and the projected values. These tables are used to assess the efficiency of different farms in terms of cost reduction and technical efficiency. Let's break down the interpretation for each table:

 Table 6:

 Each row in Table 6 corresponds to a different farm.

 The "Original movement" represents the initial cost or expenditure for various inputs in each farm.

 The "Radial movement" indicates how much a farm can reduce its costs while maintaining a similar level of production.

 The "Slack value" represents the excess or unutilized resources.

 The "Projected" column shows the projected cost after optimizing.

 Table 7 and Table 8:

 These tables follow a similar format to Table 6, providing information for additional farms.

 These tables are crucial for evaluating farm efficiency. Farms that can reduce their costs and have lower slack values are generally considered more efficient in terms of resource utilization. The "Projected" column indicates the expected cost once these efficiencies are realized. The tables enable a comparative analysis of different farms to identify areas where cost reduction and efficiency improvements can be made.

 To identify the best farm, we should consider the one with the most favorable projection values, which reflect reduced costs and increased efficiency. Specifically, we are looking for farms with the following characteristics:

 Low Slack Values: Farms with lower slack values have fewer unutilized resources and, therefore, are more efficient in resource allocation.

 Positive Radial Movement: Positive radial movement means that the farm can reduce its costs while maintaining similar production levels, which is a sign of efficiency improvement.

 Low Projected Costs: The lower the projected cost, the more efficient the farm in terms of cost reduction.

 If you have specific questions or need further analysis of the data from these tables, please feel free to ask.  The analysis and interpretation go before of this Tables 6, 7 and 8. A resume go in the subsection Input projection.

 [7] Conclusion.  The authors concluded: “This study used DEA to investigate the efficiencies of Tlaxcala’s dairy farm for data from 102 farmers in 2020. Using the VRS model and multi-stage method the efficiency of the Tlaxcala dairy farm was assessed.”  It is unclear what is meant by “multi-stage” here.  In DEA analysis, multi-stage has a specific connotation: stage 1: calculating DEA scores (efficiency scores) and stage 2: application of statistical methods to explain the efficiency scores, using, in this case, farm characteristics obtained through the survey.  Clearly, stage 2 was not conducted in this study.  Therefore, the claim that multi-stage was applied was rather confusing.

**Response:**

 The observation regarding the ambiguity surrounding the term "multi-stage" in the conclusion is noted. To clarify, in the context of this study, the multi-stage approach refers to the two distinct stages of input-oriented DEA analysis. Also we add the Bootstrap technique.

 Scale Assumption: The study adhered to the constant returns to scale (CRS) assumption in its input-oriented DEA analysis.

 Slacks Calculation: The multi-stage process involved the following:

 Stage 1: Calculating DEA scores (efficiency scores), which are clearly presented in Table 3.

 Stage 2: Application of statistical methods to explain the efficiency scores, specifically focusing on slacks. The detailed results for this stage are available in Tables 4, 5, and 6.  It considered as: Summary of output slacks, Summary of input Slacks, Summary of Peers, Summary of peer weights, Peer count summary, Summary of output targets, Summary of input target and results for each farm (variable, original, radial movement, slack movement, Slack and projected value).

 It is important to highlight that the software used for this analysis was DEAP version 2.1. This clarification aims to address any confusion regarding the application of the multi-stage method in the context of the study's DEA analysis.

 [8] References

 1. Gelan A, Muriithi B: Measuring and explaining technical efficiency of dairy farms: a case study of smallholder farms in East Africa. Agrekon. 2012; 51 (2): 53-74 Publisher Full Text

**Response:**  This references was added,(Ref. # 61).
